# Barriers and facilitators for scaling up mental health and psychosocial support interventions in low- and middle-income countries for populations affected by humanitarian crises: a systematic review

**DOI:** 10.1186/s13033-020-00431-1

**Published:** 2021-01-07

**Authors:** Jordan Troup, Daniela C. Fuhr, Aniek Woodward, Egbert Sondorp, Bayard Roberts

**Affiliations:** 1 Faculty of Public Health and Policy, Department of Health Services Research and Policy, London School of Hygiene and Tropical Medicine, 15-17 Tavistock Place, London, UK; 2 KIT Health, KIT Royal Tropical Institute, Mauritskade 64, Amsterdam, The Netherlands

**Keywords:** Mental health and psychosocial support, Humanitarian crises, Scaling up

## Abstract

**Background:**

Humanitarian crises increase the burden of mental disorders due to exposure to traumatic events and ongoing daily stressors. Effective mental health and psychosocial support (MHPSS) interventions exist, but barriers and facilitators for scaling up those interventions are less understood. The study aim was to identify barriers and facilitators for scaling up MHPSS interventions for populations affected by humanitarian crises in low- and middle-income countries.

**Methods:**

A systematic review following PRISMA guidelines was conducted. Types of scale up were summarised, and barriers and facilitators analysed using the World Health Organization’s Expandnet framework of scaling up. Evidence quality was appraised using the Mixed Methods Appraisal Tool.

**Results:**

Fourteen eligible studies were identified. Most described horizontal types of scale up, integrating services into primary and community care through staff training, task-sharing, and establishing referral and supervision mechanisms. Barriers were reported in a range of framework elements, but primarily related to those in the health system. The overall quality of studies were limited.

**Conclusion:**

Few MHPSS interventions in humanitarian crises appear to have been scaled up, and scaling up efforts were largely horizontal which challenges long-term sustainability. Greater focus should be on both horizontal and vertical scaling up, which should be accompanied by higher quality research.

## Background

Over 170 million people worldwide are currently affected by armed conflict, with the vast majority in low- and middle-income countries (LMICs) [1]. This includes over 70 million individuals forcibly displaced of which forty one million people are internally displaced (IDPs), while others have crossed international borders as refugees and asylum seekers [2]. Given the protracted nature of many conflicts, these populations often remain displaced for years, with the average length of displacement approximately 17 years [2]. These individuals are at higher risk of psychological problems and have a greater chance of falling in the treatment gap due to the scarcity of mental health services in LMICs [3–5]. Recent estimates indicate a prevalence of mental disorders among populations affected by armed conflict of 22%, twice as high as in non-conflict-affected populations [6]. This higher burden is due to past and current exposure to violent and traumatic events and ongoing daily stressors, including loss of livelihoods, impoverishment, social isolation, and forced displacement [7, 8].

Humanitarian crises can disrupt existing health services through the erosion of facilities, reductions in staff, supplies and medicines, and impeded access [9]. Conversely, services can increase in post-crisis situations and forced displacement settings as humanitarian agencies establish new services. However, such responses are often characterised by a lack of coordination between actors in the humanitarian field and the formal health system, often leading to the development of parallel systems of government and humanitarian agency responses [10]. In addition, humanitarian crises can increase demand for MHPSS services by elevated mental health needs among crisis-affected populations. Such populations may also face barriers to accessing care, including culturally inappropriate treatments, stigma and discrimination, limited availability of services, and language barriers [11, 12].

The evidence base and implementation of effective interventions for crisis-affected populations addressing supply and demand side barriers has grown [13–16]. This has been accompanied by guidelines which recommend multi-level, multi-sectoral Mental Health and Psychosocial Support (MHPSS) integrated at primary healthcare or community levels [17]. However, high-levels of unmet need and a large treatment gap for MHPSS services have been reported among conflict-affected populations [4].

Recommendations for addressing unmet mental health needs and the treatment gap include ‘scaling up’ mental health services [18]. Scaling up can be defined as “deliberate efforts to increase the impact of health service innovations successfully tested so as to benefit more people and to foster policy and programme development on a lasting basis” [19]. The World Health Organization (WHO) has developed guidelines to inform practice [19] and developed the ‘ExpandNet’ conceptual framework of scaling up [20]. This framework considers the evidence-based ‘innovation’ being taken to scale in the context of four elements; the resource team, user organisations, scale up strategies, and the environment. The resource team developed the innovation or promotes its wider use, whilst user organisations intend to adopt and scale up the innovation. The framework also outlines ‘type’ of scale up as either spontaneous or guided. Guided scale up can be horizontal (where innovations are replicated to serve new populations or get expanded to new geographical areas), vertical (where policies or legal action are used to institutionalise innovations into regulatory frameworks), or diversification (where new innovations are added to existing interventions). The framework also outlines key scale up strategies. Dissemination describes the methods chosen to transfer the innovation, such as training. Other strategies include organisational choices such as centralised or decentralised approaches, cost and resource mobilisation strategies (e.g. cost assessments), and monitoring and evaluation strategies (e.g. local needs assessments and situational analyses). The framework also highlights the need to assess opportunities and barriers for scaling up within the environment (i.e. conditions external to the user organisation). These include policy/politics; bureaucratic factors inside institutions and organisations; health sector characteristics such as leadership, reforms, and the general structure of the system; socioeconomic and cultural factors of the society; and people’s needs and rights [19].

Whilst there has been progress in scaling up services for a number of global health priorities [21, 22], this progress has been slower within the field of mental health [18]. Reported challenges to scaling up mental health services among the general population in LMICs include financial and human resource constraints, the low priority accorded to mental health by policy makers, the challenge of changing poorly organised services (e.g. over-centralised care), and poor management or leadership [18, 23–25]. However, there is less understanding on scaling-up MHPSS specifically for crisis-affected populations in LMICs. This is required given the elevated levels of mental disorders among crisis-affected populations, the particular stressors experienced by crisis-affected populations, and specific characteristics of health system responses in humanitarian situations.

This paper aims to examine barriers and facilitators for scaling up MHPSS interventions for populations affected by humanitarian crises in LMICs, in order to inform future scale up of interventions in these settings. Specifically the review will (a) describe the types or strategies of scaling up MHPSS interventions for populations affected by humanitarian crises according to the ExpandNet framework; (b) identify factors that facilitate and impede the scale up of MHPSS interventions for populations affected by humanitarian crises; and (c) assess the strength of the evidence on scaling up MHPSS interventions for populations affected by humanitarian crises.

## Methods

A systematic review methodology was used following PRISMA reporting guidelines [26]. The PRISMA Checklist is included in Additional file 1: Appendix S1.

### Eligibility criteria

The primary outcome was the scaling up of MHPSS activities. MHPSS activities were defined as “any type of local or outside support that aims to protect or promote psychosocial well-being and/or prevent or treat mental disorder” (p.5) [17]. Papers were included if they engaged in scale up activities with the intention of scaling up MHPSS. Scaling up was conceptualised according to the WHO/ExpandNet framework mentioned above [20]. The main health outcomes of interest were mental disorders and psychosocial distress. We focused on civilian populations affected by humanitarian crisis in LMICs. Populations in humanitarian crises included refugees, IDPs, non-displaced conflict-affected persons (e.g. those remaining or entrapped in areas affected by conflict), those affected by natural disasters, and those living in post-conflict settings (defined as ten years or less after the formal end of conflict). Studies were excluded if the population comprised military veterans or health staff.

### Search terms and strategy

Published literature was searched using the Embase, Medline, PsychInfo and Global Health databases until September 2019. The following search terms were combined with each other: mental health outcomes (e.g., depression, anxiety, common mental disorders); and humanitarian populations or settings (e.g., post-conflict, natural disaster, war; and scaling up (e.g., scale up, scaling up, integration, expansion)); and mental health interventions, programmes or service delivery platforms (e.g., primary health care, MHPSS, community care). Whilst no search limits were set on language, only papers published in English were included in the screening process. The full search strategy is included in Additional file 1: Appendix S2.

### Analyses and quality appraisal

A systematic narrative synthesis approach was used to summarise types of scaling up and barriers and facilitators. Data extraction and quality appraisal was double checked by the second author (random selection of 20% of the included papers). The WHO/ExpandNet Framework [20] was used to guide the analysis and synthesis, and barriers and facilitators reported by included studies were summarised in relation to the framework’s elements: the innovation, resource team, user organisation, environmental factors, and scale up strategies [20].

The quality of included studies was assessed using the Mixed Methods Appraisal Tool (MMAT; version 2018) [27]. The MMAT allows for the appraisal of quantitative, qualitative and mixed research designs. It begins with two screening questions, followed by five sections to be completed depending on the study design. As per MMAT guidance [27] articles that included supplementary methodological information were considered as well.

## Results

**Fig. 1:**
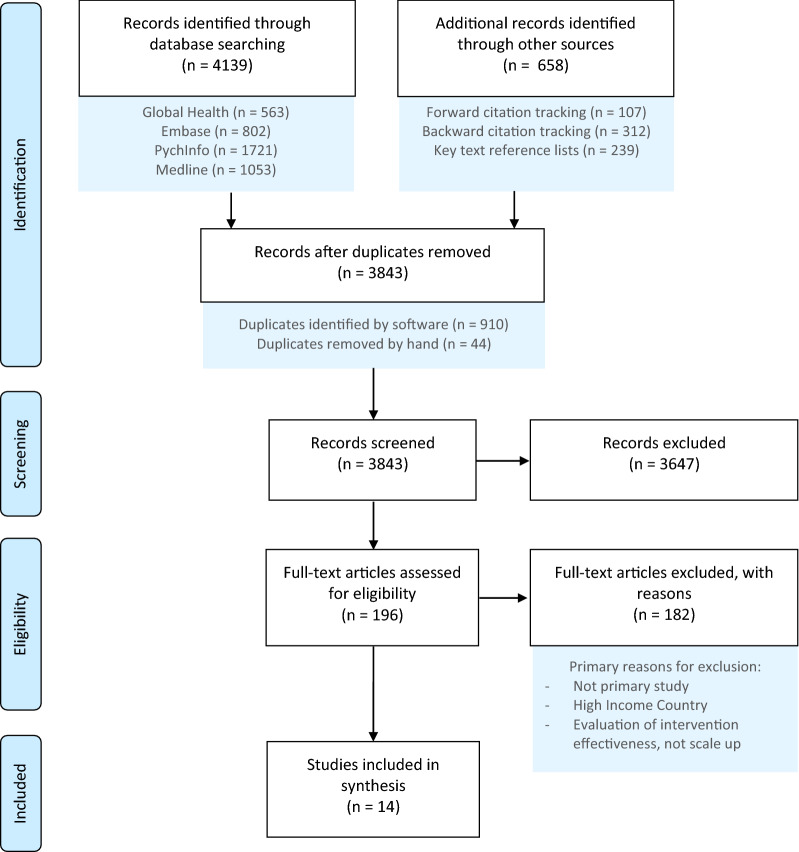
PRISMA flow diagram

A total of 4139 articles were returned by the search, with 14 meeting eligibility criteria [28–41]. Details on the screening process are provided in Fig. 1.

### Study characteristics

Study characteristics are presented in Table 1. Within the eligible 14 studies, there were 22 distinct populations targeted by scale up. Of these, eight had been affected by natural disaster [28–33] and fourteen by conflict [31, 35, 36, 38–42]. Five studies covered IDP populations [28, 30, 32, 37, 41], nine refugee populations [31, 35, 36], and 12 were local (non-displaced crisis-affected) populations [29–34, 36, 38–41].

**Table 1:** Study characteristics

First author, year	Country (classification^a^) Crisis population		Resource team		User organisation		Innovation/Service being scaled up	Adult/child/both	Mental health outcomes targeted
Baingana, 2011	Uganda (L) Natural Disaster IDP		NGO: TPO Uganda		NGO, Community and Government health services		Recognition, assessment and management Outreach services Community response	Both	MHPSS needs; CMDs
Boothby, 2011	Indonesia (LM) Natural Disaster Local population		MoH, Indonesia University^b^, WHO, ADB, DfID AusAID, USAID		JSI, Indonesian Department of Health		Case management Community outreach Home visitation	Both	Axis I disorders (DSM-IV)
Budosan, 2007	Sri Lanka (LM) Natural Disaster Local and IDP		INGO: IMC, WHO		IMC, Divisional Medical Officers of Health		Community detection ‘Established mental health interventions’ Problem solving Treatment for medically unexplained somatic pain	Not stated	Emotional and psychological health
Budosan, 2011	1: Sri Lanka (LM) Natural Disaster Local population 2: Pakistan (L) Natural Disaster Local population 3: Jordan (LM) Conflict Refugees		INGO: IMC		IMC, MoH, WHO, district staff		Community detection ‘Established mental health interventions’ Problem solving strategies Treatment for medically unexplained somatic pain	Not stated	Overall mental health
Budosan, 2011	Haiti (L) Natural Disaster Local and IDP		NGO: Cordaid		Local NGOs, Ministry of Public Health and Population, local health departments		MHPSS interventions; problem solving skills, anxiety management, anger management, family and peer support, psychological support, stress management and medication	Not stated^c^	Priority MNS conditions General mental health and wellbeing
Budosan, 2016	Philippines (LM) Natural Disaster Local population		WHO		Government, INGO: IMC		Psychosocial interventions MedicationAcute psychiatric units Psychoeducation Detection	Not stated	CMDs and stress related conditions
Chandrasiri, 2015	Sri Lanka (LM) Natural Disaster Local population		WHO		Government Staff		Identification of CMDs Crisis Intervention Community and clinic outreach Advocacy and family support Problem solving School programmes	Not stated	General mental health
Echeverri, 2018	Seven countries in Sub-Saharan Africa: Cameroon (LM), Chad (L), Ethiopia (L), DRC (L), Kenya (LM), Uganda (L), Tanzania (L) Conflict implied Refugees		UNHCR, WHO		War Trauma Foundation, UNHCR, partner organisations		Identification and care, including medication and psychosocial interventions	Not stated	MNS conditions
Hijazi, 2011	Lebanon (UM) Conflict Refugees and Local population		INGO: IMC		IMC, MoH, local partners, IDRAAC^d^, local NGO: AMEL Association		Medication Other interventions for behavioural disorders, maternal depression, monitoring child development and ADHD Psychosocial interventions including family support	Both	Range of disorders: local priorities including CMDs, severe disorders, medically unexplained symptoms, sleep, maternal mental health, and child and adolescent mental health
Humayun, 2017	Pakistan (LM) Conflict IDPs		WHO, IMC		Pakistan Army Field Hospital, IMC, District Health Office		Psychosocial interventions including: Psychoeducation Behavioural activation Stress management Problem solving Principles of behavioural therapy Counselling	Both	CMDs Priorities identified include depression, adjustment disorders, drug dependence, intellectual disabilities and behavioural disorders
Jordans, 2016	Nepal (L) Conflict Local population		Academic Institutions, NGO: TPO		NGO: TPO Nepal, Ministry of Health and Population		Assessment and management Psychoeducation, emotional support Pharmacological treatment Brief, focussed, manualised problem-oriented psychosocial support (Behavioural Activation, Motivational Interviewing, CBT) Community screening and monitoring	Not stated^e^	Local priority mental health disorders, in particular, psychoses, depression, alcohol use disorders, epilepsy
Sadiq, 2011	Iraq (LM) Conflict Local population		MoH, INGO: IMC		MoH		Assessment, diagnosis and management	Not stated	Mental health, common neurological disorders, psychiatric disorders
Shackman, 2013	Sierra Leone (L) Conflict Local population		INGO: CAFOD		Five local NGOs, University of Makeni		Counselling Family Support Medication ‘Intervention’ Awareness and community sensitisation Livelihood support	Not stated	General mental health
Siriwardhana, 2016	Sri Lanka (LM) Conflict Local population and IDPs		WHO, NIMH		MoH, Provincial health authorities and medical association		Identification and treatment	Not stated	CMDs: focus on depression, stress related disorders, medically unexplained symptoms, alcohol/drug use disorders and suicide

*TPO* Transcultural Psychosocial Organisation; *ADB* Asian Development Bank; *DfID* UK Department for International Development; *AusAID* Australian Government Overseas Aid Program (AusAID) ; *USAID* United States Agency for International Development; *JSI* John Snow Inc.; IMC = International Medical Corps; *MNS* Mental, Neurological and Substance Use; *CMD* Common Mental Disorders; *MoH* Ministry of Health; *UNHCR* United Nations High Commissioner for Refugees; *NIMH* National Institute of Mental Health; *CAFOD* Catholic Agency for Overseas Development (CAFOD)

^a^Classification according to the World Bank at the time of the study

^b^University Faculty of Nursing

^c^Mention of ‘effects of extreme stressors on children and adolescents’ in PHC training topics (not community training)

^d^Lebanese Institute for Development Research Advocacy and Applied Care (IDRAAC)

^e^Some services users below age 18, but child and adolescent services not mentioned

Innovations covered a range of assessment, management, community and outreach services. On occasion, the innovation was clearly defined as a specific pre-existing intervention e.g., behavioural activation or motivational interviewing [38] but overall more detail was provided about training content rather than intervention. The mental health outcomes covered a range of common mental health problems, sometimes informed by baseline needs assessments included in the study [32, 38], pre-existing research on priorities within the population [41] or the pre-existing content of chosen training guidelines.

Resource teams included primarily local and international Non-Governmental Organisations (NGOs), although some also involved government and academic institutions. User organisations included a mixture of local and national NGOs but had included more commonly government ministries and health system organisations. Resource teams and user organisations sometimes overlapped.

### Types of scaling up MHPSS interventions

Table 2 presents a summary of scale up types, evaluation methods and key outcomes (see Additional file 1: Appendix S3 for additional details). All studies employed horizontal types of scale up, typically integrating mental health services into primary health care (PHC) and/or community services. This was often achieved by training existing PHC and community health staff or identifying and training new community and village workers using task-sharing approaches (i.e. delegation of tasks from mental health professionals to existing of new cadres at lower levels) and/or train-the-trainer models. Supervision mechanisms and referral pathways were frequently established [28–30, 32–38]. To overcome demand-side barriers of their interventions, some included community sensitisation, mobilisation and awareness raising activities [28, 29, 36, 40], and specific community detection mechanisms [38]. Five of the 14 studies used vertical strategies [30, 33, 36, 38, 39] such as having guidelines approved by the Ministry of Health to promote mental health policy [30], typically to support horizontal expansion.

**Table 2:**
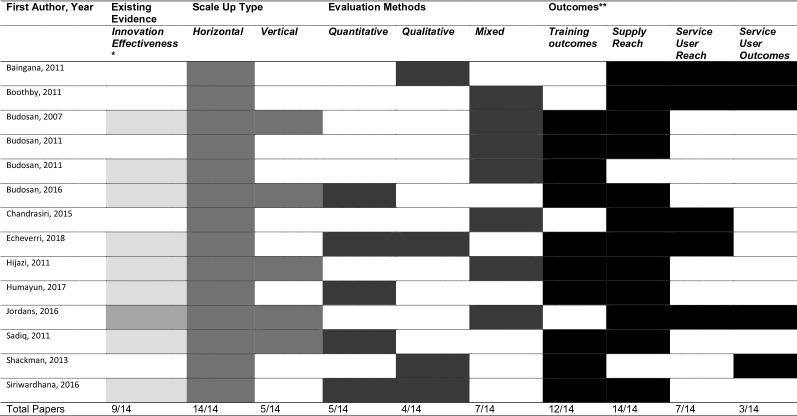
Summary of scale up types, evaluation methods and outcomes of included studies

^a^Lighter Grey = guidelines used for staff training were referenced in the article but the clinical effectiveness of the innovation being scaled up was not, Darker Grey = evidence of clinical effectiveness of innovation was referenced in article

^b^Outcome Categories were determined according to the different levels of outcomes reported across eligible studies, to provide a summary—see Additional file 1 for further study-specific details

All studies used staff training as a key scale up activity, and evaluating training effectiveness was a common evaluation method and typically completed by measuring knowledge change between pre- and post-training or field competency observations [30–33, 35–37, 39, 41]. Most studies also commented on the additional supply-side resources achieved through scaling up, such as the number of staff trained, the number of facilities with trained staff, or the ratio of trained staff to population [28–31, 33–39, 41].

### Factors that facilitate and impede the scale up of MHPSS interventions

A total of 173 barriers and 136 facilitators were identified. Table 3 summarises the number of factors reported by each study across the elements of the Framework [20].

**Table 3:**
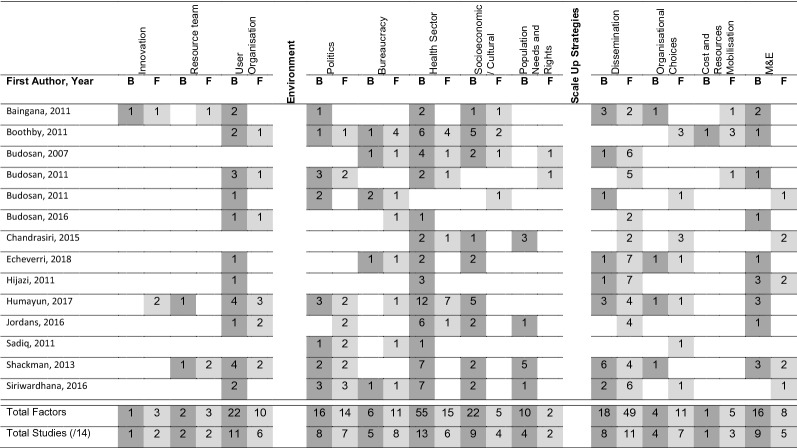
Distribution of barriers and facilitators across the WHO’s expandnet framework elements for included studies

B = Barrier (dark grey shading). F = Facilitator (light grey shading). Number in cell = number of barriers or facilitators reported by study within Framework element

#### Innovation and resource team

For innovation, the only barrier referred to was that the innovation did not target children despite the high burden among this population [28]. For the resource team, barriers to scale up included an inadequate amount of oversight and support for trained service providers [40]. The availability of ongoing technical assistance, particularly for the maintenance phase of the programme was reported as a facilitator for the resource team [29]. Another facilitator was clear strategic goals and additional oversight [40].

#### User organisations

Eleven studies reported a total of 22 user organisation barriers, including lack of skilled personnel [29, 40], high staff turnover [28, 29, 35, 41], lack of staff motivation [32, 37, 41], challenges for personnel to change practice [33, 36, 37], and competing priorities [28, 31]. Facilitators included having trained staff at managerial levels [33], experienced staff and organisations in the field [37, 38], and good coordination between staff groups [37, 40].

#### Environment: policy/politics and bureaucracy

One hundred and nine barriers to scaling up MHPSS were reported within the policy environment. Primary policy factors included lack of mental health policy promoting integration [31, 41], lack of policy implementation [31, 41], lack of political will to prioritise mental health [29, 31, 37, 40], and the distribution of financial resources as disproportionate to need [28, 37]. Specific mention of the humanitarian context was made with regards to the challenge for policy makers operating in situations of continued conflict [39] and additional security issues during election periods post-conflict [41]. Facilitators included advocacy at policy level [29, 37], programmes that are in line with policy [39, 41], and the adoption of policies specifically including integration and decentralisation of services [31, 38, 40].

Lack of awareness on MHPSS [29], consensus on scaling up [30], cooperation [32] and involvement [33, 35, 40] of policy makers and government officials in expansion of services were suggested to impede scale up. Discussions with resistant officials, appointment of mental health representatives within government, and their participation in programmes were reported to facilitate scale up. Specific characteristics of policy makers such as high motivation to improve services [33] and being a mental health professional themselves [39] also facilitated scale-up.

#### Environment: health sector

The health sector was the element in the Framework with the most reported barriers to scaling up MHPSS (55) and was reported by most studies (13/14). The large geographical catchment areas of health services resulting in challenges for staff to travel to communities and individuals to reach services [28, 29, 37], the lack of clinics and facilities [38, 40], medication shortage and centralisation [28–30, 38, 40], the lack of communication between levels within the health system [29, 36], the dearth of community and mental health services [31, 37], and the lack of resources within primary health care were reported barriers [37, 40]. Barriers regarding health professionals included the lack of human resources for mental health [30, 34, 36, 41], time constraints and workloads [30, 33, 37, 38, 41], and lack of existing mental health knowledge [30, 35, 37, 40, 41]. Additional barriers included roads being washed away contributing to remote locations becoming further isolated, and oversaturation of NGOs immediately following crisis. Facilitators included pre-existing administrative and supervisory capabilities [31, 34], and additional support including stricter two-way referral systems to support the care continuum [29].

#### Environment: socioeconomic/cultural and people’s needs and rights

Ethnic, linguistic, and religious factors were reported to further isolate certain groups within affected populations and impede MHPSS service provision [29, 35, 37]. Demand side barriers included stigma around mental health [29, 34, 38, 41], reluctance to discuss emotional difficulties [29], and a lack of family and social support [28, 37]. Factors facilitating scale up included integrated community and village workers [28, 29], sensitivity to local explanatory models [29], community participation [33], and respecting patient confidentiality [30, 31].

#### Scale up strategies: dissemination and organisational choices

Factors related to dissemination were most frequently reported amongst the four scale up strategies. Dissemination was impeded by a lack of refresher training or follow up [28, 36, 37, 40], inadequate selection criteria of the trainees [35, 40], and westernised or overly complex curricula with a lack of culturally relevant content and materials [37, 40, 41]. Conversely, factors facilitating dissemination included strategically selected trainees who were highly motivated, experienced and integrated into the community [30, 34], training that was culturally and operationally adapted to the context [30, 31, 37, 40, 41], guideline-based [30, 31], and followed up with refresher training and supervision [28, 30, 34, 35, 41]. Other recommended dissemination strategies include proactive community detection, manualising protocols to facilitate treatment termination, and delegating responsibilities within staff groups to avoid overburdening trainees [38]. Services that were integrated [32] into the existing health care system [34, 39] and addressed multiple levels of the care continuum [29] were considered facilitating organisational choices.

#### Scale up strategies: cost/resource mobilisation and monitoring and evaluation

Barriers relating to monitoring and evaluation included inconsistencies between activities and reports [28, 29, 38], poorly adapted data collection forms [33, 35], lack of pre-existing routine data and health information systems [36, 37] and paper-based systems [40]. Facilitators comprised realistic and workable documentation systems including methods for duplication and back-up [40], and a tiered, comprehensive information system particularly for staff performance monitoring [34]. Further details are available in Additional file 1: Appendix S4.

### Quality of the evidence

Six out of fourteen included studies reported “good quality” quantitative, qualitative or mixed methods research [29, 36–38, 40, 41], however a significant proportion of ratings overall were “cannot tell” (eight studies in total). Qualitative designs often failed to demonstrate that interpretations were substantiated by data and omitted critical data collection or analysis information, making the coherence of methods throughout the study difficult to ascertain. Recurrent concerns among quantitative designs were rooted in a lack of information, particularly on complete outcome data, risk of nonresponse bias, and whether the exposure/intervention/training was delivered as intended. Further details are available in Additional file 1: Appendix S5.

## Discussion

The evidence base around MHPSS interventions for populations affected by humanitarian crises has grown in recent years [15, 43]. However, the treatment gap remains high among conflict-affected populations [4], and research to support expanding MHPSS coverage has been slow [44]. Our review of the peer-reviewed literature suggests that a limited number of MHPSS interventions have been taken to scale. While our database search initially returned over 4000 articles, only 14 studies met our inclusion criteria. These criteria were already quite broad, including all kinds of MHPSS interventions and humanitarian contexts (disasters and conflict-affected, as well as immediate and more protracted situations), which highlights the dearth of evidence on scaling up MHPSS in humanitarian contexts. The popularity of the term ‘scaling up’ (and its related versions) in the published literature may explain why our initial search returned such a large number of articles.

The majority of studies within our review intended to scale up mental health interventions within PHC or community care, which is in line with current recommendations [18]. A similar approach has been taken for mental health interventions in stable LMICs [18], but research has also demonstrated that integration is less successful if the existing health system itself is not well resourced [45], which is commonly the case in LMICs and conflict-affected situations. One effect of the limited health resources has been for governments to charge health care user fees to refugees which they can clearly rarely afford given the acute poverty and lack of employment opportunities commonly experienced by refugees.

Following definitions of scale up types by WHO/ExpandNet [19], scaling up amongst included studies was predominantly horizontal as their focus was on the expansion of the innovation; however, programme activities promoting some level of integration into existing systems could be perceived as working towards vertical scaling up (e.g. identifying and training existing and new MHPSS staff, establishing referral and supervision mechanisms). Horizontal and vertical scaling up could be viewed as being on a continuum; with the increase of both required to achieve a sustainable scale up [20]. A sustainable scale up requires support by authorities, including local and national government, for ensuring that the mental health intervention is institutionalised across the country through legal and policy mechanisms allowing long-term funding and supporting the expansion of the intervention through guidelines and strategic policy documents [20]. There are positive examples of host government support for incorporating refugees into national MHPSS strategies [46]. However, the role of government is clearly extremely challenging in contexts where the government is unresponsive to refugee health care needs, is a belligerent in the conflict or has lost the trust of its citizens, which fuels the need for the development of separate humanitarian services.

MHPSS interventions reported in studies included in our review involved detection, assessment, treatment and management of mental health needs delivered by a range of village, community, primary health care and specialist staff. Evaluation methods for scale up varied and included outcomes for specific scale up activities (e.g. training effectiveness), service supply (e.g. numbers of staff trained), population reach (e.g. service users seen) and population outcomes (e.g. change in prevalence).

The high frequency and proportion of papers in our review reporting health sector barriers (e.g. lack of facilities and general mental health services, absence of human resources for mental health, and high workloads of staff) reflect the existing evidence that health system barriers significantly impede scale up if not addressed [47]. Since user organisations are commonly part of the health system, reported barriers on this element (e.g. lack of skilled staff and motivation, high staff turnover, challenges for staff to change practice) are closely linked to those in the health sector. Health system related barriers imply that mental health interventions in humanitarian crises need to be developed by taking these context-specific characteristics and fragilities of the systems into account to maximise scalability of the intervention. This can be ensured early on in the research process through adapting the intervention to the delivery systems and the populations it will serve [48]. Our review also identified a number of socio-economic and cultural factors compounded by crises that can further prevent people from accessing MHPSS services (e.g. demand side barriers including stigma and discrimination). Strategies for reducing demand-side barriers were designed in most but not all studies, and these need to be addressed if scaling up mental health interventions is to be done effectively.

Barriers and facilitators related to scale up mental health interventions to populations affected by humanitarian crises partially reflect those mentioned by other disease programmes in stable LMIC settings. For example, similarities of scale up barriers between priority health areas, such as maternal health, child health, tuberculosis, malaria and HIV/AIDS [49], and between communicable and non-communicable diseases have been highlighted [21, 22]. These include demand-side barriers, lack of human resources, inequitable availability of mental health services, referrals and linkages, and community involvement [21, 23, 50]. From these similarities, recommendations have been made to apply lessons learned to non-communicable diseases, including the use of multi-disciplinary teams, family-focussed care, engagement of stakeholders and civil society, task-sharing, community-based and home-based care, health systems strengthening and monitoring, evaluation and programme design [21]. Whilst some of these recommendations are reflected in the papers within this review, in particular community-based care and task-shifting, others are less represented and these may be more specific to humanitarian crises. For example, our findings show that existing evidence on the effectiveness of mental health interventions can promote political will to scale up, however, this was reported by a number of included studies as barrier rather than facilitator. This shows that better dissemination of findings on the intervention, and advocacy to policy makers about evidence-based mental health interventions are essential pillars of scale up. Other barriers specific to humanitarian contexts included oversaturation of user organisations (i.e. NGOs) immediately following crises including lack of coordination and implementation of short-term programmes by these organisations using inconsistent staff trainings with little engagement of national governments.

The strategy of dissemination reported the most facilitators (e.g. training and booster sessions for staff provided) of scale up strategies referred to in the articles included in this review, followed by health sector and policy factors supporting a sustainable scale up through embedding the innovation in legal and policy frameworks. Monitoring and evaluation mechanisms were also commonly reported; however, methods for measuring coverage were varied and difficult to compare. Possible explanations for this variation are the wide variety of MHPSS interventions which were reported across studies and limited consensus on outcome measures for scaling up strategies or supporting guidelines.

### Limitations

We searched four bibliographic databases and included articles which were published in English only. Grey literature was not included. It was sometimes challenging to demarcate activities of scaling up from implementation research efforts as the language used to describe them commonly overlaps. Efforts were made to be inclusive during the search strategy by including a range of related search terms for scaling up. We note that using the WHO/ExpandNet Framework [20] to synthesise results rather than taking a bottom-up approach may have lost some of the complexity of results. However, the Framework provides a widely used and comparable model within which to categorise factors.

## Conclusion

We found limited evidence in the peer-reviewed literature that MHPSS interventions for populations affected by humanitarian crises have been scaled up, and the quality of studies was limited. The WHO Expandet framework for scaling up was useful as overall theoretical framework which guided the synthesis of our findings. Our results showed that scaling up efforts were largely horizontal which challenges long-term sustainability of new programmes. Increased efforts should be made to integrate MHPSS interventions into existing delivery systems, following principles of vertical scaling up. Further research of a more rigorous quality is required, reporting in more detail on humanitarian context specific facilitators and barriers to scaling-up.

## Supplementary Information

**Additional file 1: Appendix S1.** PRISMA Checklist. **Appendix S2.** Full Search Strategy for MedLine. **Appendix S3.** Scale Up Types and Activities, Evaluation Methods and Outcomes. **Appendix S4.** Full Table of Reported Barriers and Facilitators. **Appendix S5.** Quality Appraisal of Included Studies using the Mixed Methods Quality Appraisal Tool (MMAT)

## Data Availability

The data and materials used for the current study are available from the corresponding author on reasonable request.

## Abbreviations

IDPs: Internally displaced persons
LMIC: Low- and middle-income country
MHPSS: Mental Health and Psychosocial Support
WHO: World Health Organization
