# Supplementary material for: Barriers and facilitators for scaling up mental health and psychosocial support interventions in low- and middle-income countries for populations affected by humanitarian crises: a systematic review
Source: Int J Ment Health Syst. 2021 Jan 7;15:5. doi: 10.1186/s13033-020-00431-1 (PMC7792016; doi:10.1186/s13033-020-00431-1)
Supplement: Supplementary file 1 — Additional file 1: Appendix S1. PRISMA Checklist. Appendix S2. Full Search Strategy for MedLine. Appendix S3. Scale Up Types and Activities, Evaluation Methods and Outcomes. Appendix S4. Full Table of Reported Barriers and Facilitators. Appendix S5. Quality Appraisal of Included Studies using the Mixed Methods Quality Appraisal Tool (MMAT) [file 13033_2020_431_MOESM1_ESM.docx]

Appendices

# Appendix S1: PRISMA Checklist

| **Section/topic** | **#** | **Checklist item** | **Reported on page #** |
| --- | --- | --- | --- |
| **TITLE** | | |  |
| Title | 1 | Identify the report as a systematic review, meta-analysis, or both. | 1 |
| **ABSTRACT** | | |  |
| Structured summary | 2 | Provide a structured summary including, as applicable: background; objectives; data sources; study eligibility criteria, participants, and interventions; study appraisal and synthesis methods; results; limitations; conclusions and implications of key findings; systematic review registration number. | 6 |
| **INTRODUCTION** | | |  |
| Rationale | 3 | Describe the rationale for the review in the context of what is already known. | 7-10 |
| Objectives | 4 | Provide an explicit statement of questions being addressed with reference to participants, interventions, comparisons, outcomes, and study design (PICOS). | 11 |
| **METHODS** | | |  |
| Protocol and registration | 5 | Indicate if a review protocol exists, if and where it can be accessed (e.g., Web address), and, if available, provide registration information including registration number. | 12,52-60 |
| Eligibility criteria | 6 | Specify study characteristics (e.g., PICOS, length of follow-up) and report characteristics (e.g., years considered, language, publication status) used as criteria for eligibility, giving rationale. | 12-14 |
| Information sources | 7 | Describe all information sources (e.g., databases with dates of coverage, contact with study authors to identify additional studies) in the search and date last searched. | 14 |
| Search | 8 | Present full electronic search strategy for at least one database, including any limits used, such that it could be repeated. | 14,61-65 |
| Study selection | 9 | State the process for selecting studies (i.e., screening, eligibility, included in systematic review, and, if applicable, included in the meta-analysis). | 14 |
| Data collection process | 10 | Describe method of data extraction from reports (e.g., piloted forms, independently, in duplicate) and any processes for obtaining and confirming data from investigators. | 14 |
| Data items | 11 | List and define all variables for which data were sought (e.g., PICOS, funding sources) and any assumptions and simplifications made. | 14,67 |
| Risk of bias in individual studies | 12 | Describe methods used for assessing risk of bias of individual studies (including specification of whether this was done at the study or outcome level), and how this information is to be used in any data synthesis. | 15 |
| Summary measures | 13 | State the principal summary measures (e.g., risk ratio, difference in means). | 15 |
| Synthesis of results | 14 | Describe the methods of handling data and combining results of studies, if done, including measures of consistency (e.g., I^2^) for each meta-analysis. | 15 |
| Risk of bias across studies | 15 | Specify any assessment of risk of bias that may affect the cumulative evidence (e.g., publication bias, selective reporting within studies). | **-** |
| Additional analyses | 16 | Describe methods of additional analyses (e.g., sensitivity or subgroup analyses, meta-regression), if done, indicating which were pre-specified. | NA |
| **RESULTS** | | | |
| Study selection | 17 | Give numbers of studies screened, assessed for eligibility, and included in the review, with reasons for exclusions at each stage, ideally with a flow diagram. | 16-17 |
| Study characteristics | 18 | For each study, present characteristics for which data were extracted (e.g., study size, PICOS, follow-up period) and provide the citations. | 18-21 |
| Risk of bias within studies | 19 | Present data on risk of bias of each study and, if available, any outcome level assessment (see item 12). | 30-32 |
| Results of individual studies | 20 | For all outcomes considered (benefits or harms), present, for each study: (a) simple summary data for each intervention group (b) effect estimates and confidence intervals, ideally with a forest plot. | 22-25 |
| Synthesis of results | 21 | Present results of each meta-analysis done, including confidence intervals and measures of consistency. | 22-30* |
| Risk of bias across studies | 22 | Present results of any assessment of risk of bias across studies (see Item 15). | 32 |
| Additional analysis | 23 | Give results of additional analyses, if done (e.g., sensitivity or subgroup analyses, meta-regression [see Item 16]). | - |
| **DISCUSSION** | | | |
| Summary of evidence | 24 | Summarize the main findings including the strength of evidence for each main outcome; consider their relevance to key groups (e.g., healthcare providers, users, and policy makers). | 33 |
| Limitations | 25 | Discuss limitations at study and outcome level (e.g., risk of bias), and at review-level (e.g., incomplete retrieval of identified research, reporting bias). | 38 |
| Conclusions | 26 | Provide a general interpretation of the results in the context of other evidence, and implications for future research. | 33-39 |
| **FUNDING** | | | |
| Funding | 27 | Describe sources of funding for the systematic review and other support (e.g., supply of data); role of funders for the systematic review. | - |

**A meta-analysis was not conducted; instead, these pages refer to synthesis of results according to the methods outlined elsewhere in this report.*

# Appendix S2 – Full Search Strategy for MedLine

Conducted 04.09.2019

Database(s): **Ovid MEDLINE(R) and Epub Ahead of Print, In-Process & Other Non-Indexed Citations and Daily** 1946 to September 03, 2019
Search Strategy:

| **#** | **Searches** | **Results** |
| --- | --- | --- |
| 1 | disasters/ or emergencies/ or emergency shelter/ or exp natural disasters/ or relief work/ or rescue work/ or wildfires/ | 72609 |
| 2 | disaster*.mp. | 40499 |
| 3 | (humanitarian adj2 (cris?s or relief or response or agenc* or setting or emergenc*)).mp. [mp=title, abstract, original title, name of substance word, subject heading word, floating sub-heading word, keyword heading word, organism supplementary concept word, protocol supplementary concept word, rare disease supplementary concept word, unique identifier, synonyms] | 1051 |
| 4 | (disaster adj3 (relief or plan or response or natural or victim)).mp. [mp=title, abstract, original title, name of substance word, subject heading word, floating sub-heading word, keyword heading word, organism supplementary concept word, protocol supplementary concept word, rare disease supplementary concept word, unique identifier, synonyms] | 4493 |
| 5 | ((relief or aid or rescue) adj2 work).mp. [mp=title, abstract, original title, name of substance word, subject heading word, floating sub-heading word, keyword heading word, organism supplementary concept word, protocol supplementary concept word, rare disease supplementary concept word, unique identifier, synonyms] | 6180 |
| 6 | disaster victims/ or refugees/ or survivors/ | 32688 |
| 7 | (refugee* or "asylum seeker*").mp. [mp=title, abstract, original title, name of substance word, subject heading word, floating sub-heading word, keyword heading word, organism supplementary concept word, protocol supplementary concept word, rare disease supplementary concept word, unique identifier, synonyms] | 13296 |
| 8 | evacuee.mp. | 71 |
| 9 | evacuated.mp. | 3739 |
| 10 | (displace* adj2 (force* or population or person* or human)).mp. [mp=title, abstract, original title, name of substance word, subject heading word, floating sub-heading word, keyword heading word, organism supplementary concept word, protocol supplementary concept word, rare disease supplementary concept word, unique identifier, synonyms] | 3167 |
| 11 | exp "Warfare and Armed Conflicts"/ | 44997 |
| 12 | war.mp. | 44089 |
| 13 | ((armed or zone) adj2 conflict).mp. [mp=title, abstract, original title, name of substance word, subject heading word, floating sub-heading word, keyword heading word, organism supplementary concept word, protocol supplementary concept word, rare disease supplementary concept word, unique identifier, synonyms] | 963 |
| 14 | ("conflict affected" adj3 (population* or person* or commnunit*)).mp. [mp=title, abstract, original title, name of substance word, subject heading word, floating sub-heading word, keyword heading word, organism supplementary concept word, protocol supplementary concept word, rare disease supplementary concept word, unique identifier, synonyms] | 99 |
| 15 | avalanche*.mp. | 2926 |
| 16 | earthquake*.mp. | 8552 |
| 17 | flood*.mp. | 15780 |
| 18 | landslide*.mp. | 611 |
| 19 | avalanches/ or earthquakes/ or landslides/ or tidal waves/ or tsunamis/ or volcanic eruptions/ or wildfires/ | 5781 |
| 20 | "tidal wave*".mp. | 472 |
| 21 | tsunami*.mp. | 2538 |
| 22 | cyclonic storms/ or droughts/ or floods/ or tidal waves/ | 11058 |
| 23 | "cyclonic storm*".mp. | 1917 |
| 24 | typhoon*.mp. | 591 |
| 25 | hurricane*.mp. | 3391 |
| 26 | cyclone*.mp. | 1514 |
| 27 | drought*.mp. | 20122 |
| 28 | starvation.mp. or exp Starvation/ | 33170 |
| 29 | famine*.mp. | 2293 |
| 30 | IDP.mp. | 1299 |
| 31 | post-conflict.mp. | 740 |
| 32 | post-emergenc*.mp. | 507 |
| 33 | "internally displaced person*".mp. | 329 |
| 34 | 1 or 2 or 3 or 4 or 5 or 6 or 7 or 8 or 9 or 10 or 11 or 12 or 13 or 14 or 15 or 16 or 17 or 18 or 19 or 20 or 21 or 22 or 23 or 24 or 25 or 26 or 27 or 28 or 29 or 30 or 31 or 32 or 33 | 260014 |
| 35 | integrat*.mp. | 493753 |
| 36 | implement*.mp. [mp=title, abstract, original title, name of substance word, subject heading word, floating sub-heading word, keyword heading word, organism supplementary concept word, protocol supplementary concept word, rare disease supplementary concept word, unique identifier, synonyms] | 449299 |
| 37 | scale-up.mp. [mp=title, abstract, original title, name of substance word, subject heading word, floating sub-heading word, keyword heading word, organism supplementary concept word, protocol supplementary concept word, rare disease supplementary concept word, unique identifier, synonyms] | 10373 |
| 38 | scaling-up.mp. [mp=title, abstract, original title, name of substance word, subject heading word, floating sub-heading word, keyword heading word, organism supplementary concept word, protocol supplementary concept word, rare disease supplementary concept word, unique identifier, synonyms] | 4395 |
| 39 | coverage.mp. [mp=title, abstract, original title, name of substance word, subject heading word, floating sub-heading word, keyword heading word, organism supplementary concept word, protocol supplementary concept word, rare disease supplementary concept word, unique identifier, synonyms] | 118729 |
| 40 | accessib*.mp. [mp=title, abstract, original title, name of substance word, subject heading word, floating sub-heading word, keyword heading word, organism supplementary concept word, protocol supplementary concept word, rare disease supplementary concept word, unique identifier, synonyms] | 175729 |
| 41 | (expan* or "capacity building").mp. [mp=title, abstract, original title, name of substance word, subject heading word, floating sub-heading word, keyword heading word, organism supplementary concept word, protocol supplementary concept word, rare disease supplementary concept word, unique identifier, synonyms] | 392612 |
| 42 | 35 or 36 or 37 or 38 or 39 or 40 or 41 | 1508737 |
| 43 | MHPSS.mp. | 29 |
| 44 | ("mental health and psychosocial support" or "psychosocial support").mp. [mp=title, abstract, original title, name of substance word, subject heading word, floating sub-heading word, keyword heading word, organism supplementary concept word, protocol supplementary concept word, rare disease supplementary concept word, unique identifier, synonyms] | 3352 |
| 45 | service*.mp. | 945783 |
| 46 | program*.mp. | 946775 |
| 47 | intervention*.mp. | 958308 |
| 48 | therap*.mp. | 5837423 |
| 49 | "health system*".mp. | 47745 |
| 50 | ("primary care" or "primary health care" or "primary healthcare").mp. [mp=title, abstract, original title, name of substance word, subject heading word, floating sub-heading word, keyword heading word, organism supplementary concept word, protocol supplementary concept word, rare disease supplementary concept word, unique identifier, synonyms] | 153829 |
| 51 | community.mp. | 526885 |
| 52 | exp Mental Health Services/ or exp psychotherapy/ or health services/ or adolescent health services/ or community health services/ or community mental health services/ or emergency services, psychiatric/ or health services for persons with disabilities/ or health services for the aged/ or health services, indigenous/ or mental health recovery/ or rural health services/ or social work, psychiatric/ or occupational therapy/ or physical therapy specialty/ or capacity building/ or health communication/ or "health care quality, access, and evaluation"/ or exp "delivery of health care"/ or exp health services research/ or public health systems research/ | 1338790 |
| 53 | exp Psychotherapy, Group/ | 26155 |
| 54 | exp Primary Health Care/ | 149128 |
| 55 | "safe space".mp. | 212 |
| 56 | "mental health care".mp. | 10571 |
| 57 | "family care".mp. | 1597 |
| 58 | "family support".mp. | 5031 |
| 59 | psychoeducation.mp. | 2686 |
| 60 | Family Therapy/ | 8686 |
| 61 | "trauma healing".mp. | 41 |
| 62 | psychotherapy.mp. | 82269 |
| 63 | ((brief or low-intensity) and (CBT or cognitive behavio?ral therapy or intervention or therapy or psychotherapy)).mp. [mp=title, abstract, original title, name of substance word, subject heading word, floating sub-heading word, keyword heading word, organism supplementary concept word, protocol supplementary concept word, rare disease supplementary concept word, unique identifier, synonyms] | 48005 |
| 64 | 43 or 44 or 45 or 46 or 47 or 48 or 49 or 50 or 51 or 52 or 53 or 54 or 55 or 56 or 57 or 58 or 59 or 60 or 61 or 62 or 63 | 8341624 |
| 65 | (integrat* or implement* or scale-up or scaling-up or coverage or accessib* or (expan* or "capacity building")).mp. adj5 ((MHPSS or ("mental health and psychosocial support" or "psychosocial support") or service* or program* or intervention* or therap* or "health system*" or ("primary care" or "primary health care" or "primary healthcare") or community).mp. or (exp Mental Health Services/ or exp psychotherapy/ or health services/ or adolescent health services/ or community health services/ or community mental health services/ or emergency services, psychiatric/ or health services for persons with disabilities/ or health services for the aged/ or health services, indigenous/ or mental health recovery/ or rural health services/ or social work, psychiatric/ or occupational therapy/ or physical therapy specialty/ or capacity building/ or health communication/ or "health care quality, access, and evaluation"/ or exp "delivery of health care"/ or exp health services research/ or public health systems research/) or exp Psychotherapy, Group/ or exp Primary Health Care/ or "safe space".mp. or "mental health care".mp. or "family care".mp. or "family support".mp. or psychoeducation.mp. or Family Therapy/ or "trauma healing".mp. or psychotherapy.mp. or ((brief or low-intensity) and (CBT or cognitive behavio?ral therapy or intervention or therapy or psychotherapy)).mp.) | 250280 |
| 66 | exp Mental Disorders/ or "mental disorder*".mp. | 1199950 |
| 67 | mental.mp. | 514880 |
| 68 | psychosocial.mp. | 91966 |
| 69 | psychological.mp. | 463197 |
| 70 | depress*.mp. | 518049 |
| 71 | PTSD.mp. | 22910 |
| 72 | "post-traumatic stress disorder*".mp. | 10655 |
| 73 | neurotic.mp. | 21939 |
| 74 | neuros?s.mp. | 10569 |
| 75 | anxiety.mp. | 220814 |
| 76 | anxious.mp. | 15718 |
| 77 | (schizophreni* or psychos?s).mp. [mp=title, abstract, original title, name of substance word, subject heading word, floating sub-heading word, keyword heading word, organism supplementary concept word, protocol supplementary concept word, rare disease supplementary concept word, unique identifier, synonyms] | 175524 |
| 78 | schizothyme.mp. [mp=title, abstract, original title, name of substance word, subject heading word, floating sub-heading word, keyword heading word, organism supplementary concept word, protocol supplementary concept word, rare disease supplementary concept word, unique identifier, synonyms] | 1 |
| 79 | mania.mp. [mp=title, abstract, original title, name of substance word, subject heading word, floating sub-heading word, keyword heading word, organism supplementary concept word, protocol supplementary concept word, rare disease supplementary concept word, unique identifier, synonyms] | 10195 |
| 80 | manic.mp. [mp=title, abstract, original title, name of substance word, subject heading word, floating sub-heading word, keyword heading word, organism supplementary concept word, protocol supplementary concept word, rare disease supplementary concept word, unique identifier, synonyms] | 11137 |
| 81 | delusion*.mp. [mp=title, abstract, original title, name of substance word, subject heading word, floating sub-heading word, keyword heading word, organism supplementary concept word, protocol supplementary concept word, rare disease supplementary concept word, unique identifier, synonyms] | 13528 |
| 82 | OCD.mp. [mp=title, abstract, original title, name of substance word, subject heading word, floating sub-heading word, keyword heading word, organism supplementary concept word, protocol supplementary concept word, rare disease supplementary concept word, unique identifier, synonyms] | 9055 |
| 83 | "obsessive-compulsive disorder".mp. [mp=title, abstract, original title, name of substance word, subject heading word, floating sub-heading word, keyword heading word, organism supplementary concept word, protocol supplementary concept word, rare disease supplementary concept word, unique identifier, synonyms] | 17947 |
| 84 | phobia.mp. [mp=title, abstract, original title, name of substance word, subject heading word, floating sub-heading word, keyword heading word, organism supplementary concept word, protocol supplementary concept word, rare disease supplementary concept word, unique identifier, synonyms] | 7902 |
| 85 | phobic.mp. [mp=title, abstract, original title, name of substance word, subject heading word, floating sub-heading word, keyword heading word, organism supplementary concept word, protocol supplementary concept word, rare disease supplementary concept word, unique identifier, synonyms] | 12275 |
| 86 | somatic.mp. [mp=title, abstract, original title, name of substance word, subject heading word, floating sub-heading word, keyword heading word, organism supplementary concept word, protocol supplementary concept word, rare disease supplementary concept word, unique identifier, synonyms] | 108112 |
| 87 | somatoform.mp. [mp=title, abstract, original title, name of substance word, subject heading word, floating sub-heading word, keyword heading word, organism supplementary concept word, protocol supplementary concept word, rare disease supplementary concept word, unique identifier, synonyms] | 10462 |
| 88 | suicid*.mp. [mp=title, abstract, original title, name of substance word, subject heading word, floating sub-heading word, keyword heading word, organism supplementary concept word, protocol supplementary concept word, rare disease supplementary concept word, unique identifier, synonyms] | 90177 |
| 89 | dementia*.mp. [mp=title, abstract, original title, name of substance word, subject heading word, floating sub-heading word, keyword heading word, organism supplementary concept word, protocol supplementary concept word, rare disease supplementary concept word, unique identifier, synonyms] | 119739 |
| 90 | alzheimer*.mp. [mp=title, abstract, original title, name of substance word, subject heading word, floating sub-heading word, keyword heading word, organism supplementary concept word, protocol supplementary concept word, rare disease supplementary concept word, unique identifier, synonyms] | 150295 |
| 91 | epilep*.mp. [mp=title, abstract, original title, name of substance word, subject heading word, floating sub-heading word, keyword heading word, organism supplementary concept word, protocol supplementary concept word, rare disease supplementary concept word, unique identifier, synonyms] | 156115 |
| 92 | "alcohol use".mp. [mp=title, abstract, original title, name of substance word, subject heading word, floating sub-heading word, keyword heading word, organism supplementary concept word, protocol supplementary concept word, rare disease supplementary concept word, unique identifier, synonyms] | 32760 |
| 93 | "alcohol misuse".mp. [mp=title, abstract, original title, name of substance word, subject heading word, floating sub-heading word, keyword heading word, organism supplementary concept word, protocol supplementary concept word, rare disease supplementary concept word, unique identifier, synonyms] | 2512 |
| 94 | "alcohol abuse".mp. [mp=title, abstract, original title, name of substance word, subject heading word, floating sub-heading word, keyword heading word, organism supplementary concept word, protocol supplementary concept word, rare disease supplementary concept word, unique identifier, synonyms] | 14718 |
| 95 | psychotic.mp. [mp=title, abstract, original title, name of substance word, subject heading word, floating sub-heading word, keyword heading word, organism supplementary concept word, protocol supplementary concept word, rare disease supplementary concept word, unique identifier, synonyms] | 65213 |
| 96 | affective.mp. [mp=title, abstract, original title, name of substance word, subject heading word, floating sub-heading word, keyword heading word, organism supplementary concept word, protocol supplementary concept word, rare disease supplementary concept word, unique identifier, synonyms] | 67119 |
| 97 | mood.mp. [mp=title, abstract, original title, name of substance word, subject heading word, floating sub-heading word, keyword heading word, organism supplementary concept word, protocol supplementary concept word, rare disease supplementary concept word, unique identifier, synonyms] | 78751 |
| 98 | "child behavio?r".mp. [mp=title, abstract, original title, name of substance word, subject heading word, floating sub-heading word, keyword heading word, organism supplementary concept word, protocol supplementary concept word, rare disease supplementary concept word, unique identifier, synonyms] | 39699 |
| 99 | "common mental disorder".mp. [mp=title, abstract, original title, name of substance word, subject heading word, floating sub-heading word, keyword heading word, organism supplementary concept word, protocol supplementary concept word, rare disease supplementary concept word, unique identifier, synonyms] | 528 |
| 100 | CMD.mp. [mp=title, abstract, original title, name of substance word, subject heading word, floating sub-heading word, keyword heading word, organism supplementary concept word, protocol supplementary concept word, rare disease supplementary concept word, unique identifier, synonyms] | 2189 |
| 101 | "mental trauma".mp. [mp=title, abstract, original title, name of substance word, subject heading word, floating sub-heading word, keyword heading word, organism supplementary concept word, protocol supplementary concept word, rare disease supplementary concept word, unique identifier, synonyms] | 73 |
| 102 | stress.mp. [mp=title, abstract, original title, name of substance word, subject heading word, floating sub-heading word, keyword heading word, organism supplementary concept word, protocol supplementary concept word, rare disease supplementary concept word, unique identifier, synonyms] | 860443 |
| 103 | well-being.mp. [mp=title, abstract, original title, name of substance word, subject heading word, floating sub-heading word, keyword heading word, organism supplementary concept word, protocol supplementary concept word, rare disease supplementary concept word, unique identifier, synonyms] | 69869 |
| 104 | wellbeing.mp. [mp=title, abstract, original title, name of substance word, subject heading word, floating sub-heading word, keyword heading word, organism supplementary concept word, protocol supplementary concept word, rare disease supplementary concept word, unique identifier, synonyms] | 13933 |
| 105 | "psychological resilience".mp. [mp=title, abstract, original title, name of substance word, subject heading word, floating sub-heading word, keyword heading word, organism supplementary concept word, protocol supplementary concept word, rare disease supplementary concept word, unique identifier, synonyms] | 472 |
| 106 | "mental resilience".mp. [mp=title, abstract, original title, name of substance word, subject heading word, floating sub-heading word, keyword heading word, organism supplementary concept word, protocol supplementary concept word, rare disease supplementary concept word, unique identifier, synonyms] | 44 |
| 107 | "psychological recovery".mp. [mp=title, abstract, original title, name of substance word, subject heading word, floating sub-heading word, keyword heading word, organism supplementary concept word, protocol supplementary concept word, rare disease supplementary concept word, unique identifier, synonyms] | 257 |
| 108 | "psychosocial resilience".mp. [mp=title, abstract, original title, name of substance word, subject heading word, floating sub-heading word, keyword heading word, organism supplementary concept word, protocol supplementary concept word, rare disease supplementary concept word, unique identifier, synonyms] | 36 |
| 109 | "post-traumatic stress".mp. [mp=title, abstract, original title, name of substance word, subject heading word, floating sub-heading word, keyword heading word, organism supplementary concept word, protocol supplementary concept word, rare disease supplementary concept word, unique identifier, synonyms] | 11972 |
| 110 | "acute stress".mp. [mp=title, abstract, original title, name of substance word, subject heading word, floating sub-heading word, keyword heading word, organism supplementary concept word, protocol supplementary concept word, rare disease supplementary concept word, unique identifier, synonyms] | 6712 |
| 111 | "post-traumatic growth".mp. [mp=title, abstract, original title, name of substance word, subject heading word, floating sub-heading word, keyword heading word, organism supplementary concept word, protocol supplementary concept word, rare disease supplementary concept word, unique identifier, synonyms] | 356 |
| 112 | exp emotional adjustment/ or exp posttraumatic growth, psychological/ or exp behavioral symptoms/ or child behavior/ or problem behavior/ or drinking behavior/ or exp alcohol drinking/ or exp drug-seeking behavior/ or exp "marijuana use"/ or exp risk-taking/ | 462520 |
| 113 | mental health/ or resilience, psychological/ | 39196 |
| 114 | Dementia/ | 48203 |
| 115 | 66 or 67 68 or 69 or 70 or 71 or 72 or 73 or 74 or 75 or 76 or 77 or 78 or 79 or 80 or 81 or 82 or 83 or 84 or 85 or 86 or 87 or 88 or 89 or 90 or 91 or 92 or 93 or 94 or 95 or 96 or 97 or 98 or 99 or 100 or 101 or 102 or 103 or 104 or 105 or 106 or 107 or 108 or 109 or 110 or 111 or 112 or 113 or 114 | 3213757 |
| 116 | 34 and 65 and 115 | 1053 |

# Appendix S3. Scale Up Types and Activities, Evaluation Methods and Outcomes

| **Study Details** | **Existing evidence** | | **Scale Up** |  | **Evaluation** |  |
| --- | --- | --- | --- | --- | --- | --- |
|  | ***E^A^*** | ***CE^B^*** | ***Type***  ***Elements / Activities*** | | ***Type***  ***Methods*** | ***Main Outcomes*** |
| Baingana, 2011  Uganda  Natural Disaster  IDP | N | N | Horizontal   - Integrating mental health into Primary Health Care: - Trained health workers - Arranged psychiatric clinical officers or nurses to visit selected health centres monthly for outreach and supervision - Employed social workers to support Village Health Teams - Trained Village Health Team Members to mobilise communities, sensitise communities to mental health issues, and identify and refer patients. | | Qualitative   - Desk Review - Field visits for observation - Key informant Interviews: District Health Officials, Health Unit Managers, Senior Technical Staff, Project Partners - Focus Group Discussions with beneficiaries: service users, support group members, Village Health Team Members | - Patients successfully treated and satisfied with services - Number of patients at outreach clinics exceeded targets - Health units providing space for mental health clinics - Village Health Team workers identified by communities, mobilised patients, supported mental health clinics and followed patients up through home visits - Regular support groups formed - Data collected and included in Ministry of Health Information System - Regular mental health coordination meetings with stakeholders in each district - Number of hospital visits dropped for patients from areas where outreach took place |
| Boothby, 2011  Indonesia  Natural Disaster  Host | N | N | Horizontal   - Establish acute psychiatric care unit - Train Community Mental Health Nurses at subdistrict level with competency-based, classroom and field work curriculum reviewed by WHO and Indonesian psychiatrists - beginner, intermediate, advanced - Establish competency standards - Train mental health cadres to identify villagers in need of services, raise awareness and reduce stigma in the community, conduct home visitation and outreach to families requiring support - Establish referral pathways between Community Mental Health Workers and mental health cadres at village level, including midwives and village leaders | | Mixed   - *Adequacy survey:* examine implementation against output indicators across districts - key activities, training and services. One district failed implementation and so was used as naturally occurring baseline - Structured interviews and ‘group interviews’ with village, community, subdistrict, government and agency staff - Field observations - *Outcome Study:* social functioning changes for people with Axis I disorders receiving services from new health care system (one district) - Quantitative: Service users and families rated mental health and social functioning before (retrospective baseline) and after implementation - Qualitative: asked service user and families about observed changes | - *District level:* human-rights based ten bed acute unit built, equipped and staffed by trained nurses and psychiatrist - Staff support to subdistrict health clinic staff - *Subdistrict level:* target community nurse-to-population ratio achieved - Community nurses trained and competency standards established - *Village level:* Mental health cadres identified and trained - Several NGOs provided additional support to cadres for mental health promotion through women’s and children’s groups - Introduction or increased awareness of medical model of disease and treatment - Perceived statistically significant improvement of mental health among majority of households, with none worsening - Perceived changes (qualitative): less disruptive, improved quality of life, improved individual and family functioning, improved mental state, and employment - Baseline district reported frequent psychiatric admissions, security situations requiring police intervention, delusions and voice commands |
| Budosan, 2007  Sri Lanka  Natural Disaster  Host and IDP | G | N | Horizontal and Vertical   - Guidelines approved by MoH Colombo designed to promote mental health policy, the WHO’s existing health systems in tsunami-affected areas, and the WHO’s five-year mental health plan, which advocates for integration - Train-the-trainer model: training programme for Divisional Medical Officers of Health who then train PHC staff - Collaboration with local resources e.g., indigenous healers - Establish mobile clinics and supervision - Implement monitoring and evaluation - Develop case registry and mental health curriculum | | Mixed   - Structured Focus Group Discussions and In-depth interviews with key stakeholders (initial assessment and monitoring) - Local and international psychiatrists observed diagnostic specificity of medical officers - Clinical competency measured with checklist - Training evaluation form for trainees - Pre/post knowledge tests - Service user data and mental health assessment – data collection forms | - Theoretical and practical training delivered - DMOH curriculum developed - More mental health clinics opened in PHC - Treatment sessions delivered via mobile clinics and mental health clinics in PHC - 100% of mid-level staff below DMOHs were trained by DMOH staff - Good satisfaction with training - Demonstration of good communication, respect, working knowledge of referral procedures, correct diagnoses, and appropriate treatment decisions - Less satisfactory history taking, examinations and record keeping - Gains appeared resilience to security disruptions due to re-emerging conflict - Seven DMOH recommended for new MoH role Medical Officers of Mental Health in line with long-term plan for one in every district |
| Budosan, 2011  Sri Lanka, Pakistan and Jordan  Natural Disaster or Conflict  Host or Refugees | N | N | Horizontal   - Train PHC staff according to the Guidelines for International training in MHPSS interventions for Trauma Exposed Populations in Clinical and Community Settings | | Mixed  *Qualitative:*   - Desk review - Key informant interviews - Focus Group Discussions - Observation to evaluate staff skills *Quantitative:* - Multiple choice test for PHC doctors and mid-level staff before and after training - On-the-job competency checklist to evaluate doctor detection rate, diagnostic accuracy and treatment - Construct validity and internal consistency of instruments explored | - Sri Lanka: Significant improvement in knowledge and skills, satisfaction with training - Pakistan: No data on skills or knowledge. Satisfaction with training. - Jordan: Significant improvement in knowledge, data on skills and satisfaction pending at time of publication. |
| Budosan, 2011  Haiti  Natural Disaster  Host and IDP | G | N | Horizontal   - Identify, recruit and train community level workers - Train PHC workers of local NGOs - Establish referral system between communities and NGO and government health systems | | Mixed  *Needs and services assessment - Qualitative:*   - Field assessments before implementation - Semi-structured interviews with key informants - Focus Group Discussions with beneficiaries - Unstructured observation of patient encounters in PHC   *Quantitative:* Community survey, multiple choice test  *Implementation and training assessment:*   - Knowledge test before and after training - Satisfaction ratings | - Knowledge of community and PHC workers improved - All or nearly all workers considered the training very useful - No significant change in actual clinical practice of PHC workers - Lack of change in practice negatively affected planned referral system - Community workers identifying more cases appropriate for referral but PHC workers not motivated (professionally or financially) enough to offer assistance |
| Budosan, 2016  Philippines  Natural Disaster  Host | G | N | Horizontal and Vertical   - Community workers trained in psychosocial care and support for stress related conditions - Non-specialist healthcare providers trained in assessment and management of CMD and stress related conditions - Supervision in facilities established - Rural health care units, city health care units and government hospitals all involved in scale up | | Quantitative   - Availability and access of services indicator - government health units in targeted areas with trained staff monitored monthly by WHO and IMCs MHPSS team - Affordability of mental health services – PHC unit monthly medication reports - Staff competency – observation by WHO and IMC MHPSS teams | - Trained personnel demonstrate increased likelihood of proper community MHPSS provision and management of problems in general health care than untrained personnel - Majority of PHC units and regional hospitals had trained providers - All provincial hospitals had at least one doctor and nurse for assessment, treatment and management of CMDs - Increased availability and accessibility of services - Medicines more affordable and included in supply packs from MoH to PHCs - Regional, provincial and city MHPSS committees established for oversight - ‘Critical mass’ of trained staff reached to continue to develop mental health care - Functional resilience improved - Lack of confidence of non-specialised staff to assess and manage after theoretical training and for community workers to deliver treatment |
| Chandrasiri, 2015  Sri Lanka  Natural Disaster  Host | N | N | Horizontal   - Integration into PHC system - Community Support Officer (CSO) and Supervisor CSOs trained, including basic knowledge of mental health legislation in Sri Lanka, human rights, local health care delivery system, and data management - In-service and theoretical training - Monthly allowance for CSOs from WHO via MoH - Establish referral pathways, monitoring, follow-up and supervision mechanisms - Establish outreach clinic programme, community outreach programmes and school programmes - CSO survey of all households to identify people with mental health needs | | Mixed  *Quantitative:*   - Programme document review, including data on treatment initiation, objectives and activity plans - Progress reports and review meeting minutes retrieved for statistics on performance of CSOs   *Qualitative:*   - Key informant interviews with leading individuals in the programme and mental health and medical officers - Small focus group discussions with a group of CSOs and beneficiaries | - End of programme handover to PHC staff within MoH - Many CSOs absorbed into government health system as permanent employees - Half of all households surveyed by CSOs - 63% of referrals attended clinic - Not all new cases identified were referred - 15-20% of schools covered each year – unclear what activities were done - Significant number of promotion programmes conducted in community |
| Echeverri, 2018  Sub-Saharan African Countries  Post-conflict  Refugees | G | N | Horizontal   - Training for clinicians (physicians, clinical officers, general and psychiatric nurses, psychologists) - Training for community workers (refugee incentive workers, humanitarian staff) - Adapt training for community role and translate to native language - Focus on collaborative, multilevel working and context-specific referral systems - Establish supervision - Uganda only: Train-the-trainer-and-supervisor model, to then train general health staff | | Both   - ‘Mostly qualitative process evaluation of implementation’ - Desk review (including training reports) - Telephone interviews with UNHCR public health officers - Analysis of UNHCRs health information system data and questionnaires for trainees   *Field visits in Cameroon and Tanzania:*   - Focus Group Discussions with managers, coordinators, clinicians, community workers and refugees - Observation of clinical encounters and home visits - Inpatient visits, interviews with service users and families and review of clinical files - Pre/post training knowledge tests | - Perceived improvement of clinical skills - More appropriate use of medications - More psychosocial interventions for stress-related conditions - Perceived more people identified and treated - Referrals to specialists increased in some settings and decreased in others - Community psychosocial interventions higher in some settings - Improved knowledge and confidentiality - Monthly consultations for MNS conditions increased or decreased depending on country - Perceived positive attitude change, increased community awareness, increased partners involved in camp events, and decreased stigma - Supervision and referrals well established in some settings - Patient and family report improved quality of MHPSS care and satisfaction - Improved collaboration between levels of care and traditional healers - Case registration system for community and clinicians established. - Partnerships with national health authorities established - Mental health units in general hospitals established in several regions - Stakeholder coordination depending on setting |
| Hijazi, 2011  Lebanon  Post-conflict  Refugees and host | G | N | Horizontal and Vertical   - Train-the-trainers model to integrate into PHC - Training drafted by local psychologists, reviewed by international specialists, based on previous published guidelines, and informed by formative research (prevalence in PHC) - Recruit and train psychiatrists and psychologists as trainers, to then train PHC staff of local NGO - MoH involvement to certify training and plan national roll-out, and inform mental health plan - Approval and accreditation of training by Psychiatric Society and Order of Physicians - Establish advisory board - Theoretical, on-the-job and refresher training - Establish referral networks | | Mixed  *First evaluation to standardise training:*   - Focus Group Discussions with GPs and mid-level staff - In-depth interviews with two main trainers   *Second evaluation to evaluate training:*   - Focus Group Discussions on appropriate topics, experience, skills change, interest in refresher training - Knowledge and competency measures before, mid-term and after training - Competency checklist | - Identification of disorders and knowledge improved and trained staff met respective minimum competency standards - Communication and listening skills, tolerance for service users with vague complaints, awareness of mental health symptoms and ability to recognise somatisation versus organic problems improved - Some progress still needed for knowing when to refer, prescribe, how to manage, and when to discharge - Project resulted in further funding of activities, opportunities for further evaluation, and impact on national policy |
| Humayun, 2017  Pakistan  Post-conflict  IDP | G | N | Horizontal   - Train-the-trainer model; trainers trained, then non-specialists trained on the job - Establish regular mental health ‘camps’ - Establish supervision and referral system - Adapt training manual to setting - Establish community follow up - Helpline for acute emergencies | | Quantitative   - Knowledge tests before and after training | - Significant improvement in knowledge - Collaboration between public health services and humanitarian agencies - First MHPSS taskforce in the province was formed - Local services engaged and provided impetus for setting up mental health agenda for the region |
| Jordans, 2016  Nepal  Post-conflict  Host | N | N^C^ | Horizontal and Vertical   - Plan includes 12 packages over 3 levels – health organisation, facility and community - Primarily integration through task sharing   *Health facility level:*   - Service provider awareness and stigma reduction (course and integrated workshop with staff and beneficiaries) - Training on assessment and management of priority disorders   *Community level:*   - Community sensitisation and stigma reduction through organised stakeholder group interactions including beneficiaries - Convene large group community meetings for awareness raising - Introduce community informant detection tool introduced for proactive case finding - Train community counsellors in generic counselling and manualised interventions - Establish peer support groups for social support and linkages with services and resources - Establish cascading supervision system – case conferences with psychiatrist, spot checks by nurses, managerial supervision by district health administration, peer supervision for psychosocial care by community counsellors - Establish case register and implement competency measure - Stepped training for health workers and community counsellors with refresher training | | Mixed  *Formative research informing content and structure of care system:*   - Expert panel for prioritisation - Policy maker and service provider workshops for Theory of Change (ToC) - In-depth interviews and Focus Group Discussions with key stakeholders to assess perceptions and barriers to integration based on ToC - *Pilot testing to adapt and fine tune Mental Healthcare Plan:* - Routine monitoring data of service users - Evaluation questionnaires for randomly selected service users and providers - Service users who dropped out were followed up | - Majority of service users were somewhat or very satisfied on most indicators and majority would seek help again from the service - Dissatisfaction mainly for unavailability of prescribed medications and privacy (lack of private rooms in facilities) - Range of disorders detected and treated at community and facility levels - One third of staff are somewhat distressed due to perceived additional time required and need a clear mandate to perform duties, but majority were satisfied with outcomes of care - Reasons for service user drop out include medication side effects, time constraints, and medication unavailability - Ministry of Health and Population commitment for medication and time for staff training and mental healthcare delivery for duration of programme |
| Sadik, 2011  Iraq  Post-conflict  Host | G | N | Horizontal and Vertical   - Appoint lead in PHC and lead in specialised services in each governate to train staff, improve access to care, update records and enhance collaboration between PHC and specialist care - Arrange workshops for leads to discuss programme and outcomes - Appoint mental health advisor to MoH to lead integration - Translate and adapt existing guidelines - Deliver training course for PHC, including issues of policy, legislation, links between mental health and child health, reproductive health, HIV and malaria, information systems, community health works and traditional healers, and integration into annual operational plans | | Quantitative   - Knowledge, attitudes and practice test before and after training   *Random sample of clinics:*   - Trained and untrained staff evaluated with questionnaire and skills assessment - Observation of trained and unstrained staff skills by psychiatrist (blinded) during daily work - Exit interviews with five randomly selected service users from staff being evaluated | - Improved knowledge - Trained staff more likely to have excellent / some skill on approach, consultation and diagnosis than untrained staff - Service users perceive trained staff as better than untrained staff in approach, consultation, diagnosis and treatment - Service user satisfaction better in all domains for trained versus untrained staff - Differences between trained and untrained staff were smaller when reported by patients than psychiatrist assessments |
| Shackman, 2013  Sierra Leone  Post-conflict  Host | N | N | Horizontal   - Nurse training on awareness and intervention - Workshops for traditional healers, prison and police officers and social workers on awareness and sensitisation - Hospitals to set up centres to provide free medication and counselling services and family advice and support - Community outreach efforts by local justice, peace and human rights commission and radio station to promote awareness - Livelihood support provided by a charity for those who completed treatment - Administration and training initially by network of international experts and volunteers | | Qualitative   - Document review to establish objectives of evaluation - In-depth interviews and Focus Group Discussions with partner and government staff, service users and families, and community members - Corroborative data from reports and counselling centre records obtained where possible | - Mental health outcomes improved - Specific deliverables: Support / counselling, medication, awareness raising through radio, training workshops for nurses, traditional healers, police and prison staff - Nurses reported increased awareness of family pressures and their needs for support - Mental health profile raised by becoming a partner involved in national strategy, contributing to government mental health strategic policy and plan - Principle partner of future mental health capacity project |
| Siriwardhana, 2016  Sri Lanka  Post-conflict  Host and IDP | G | N | Horizontal   - Integration into PHC by task-sharing - PHC staff trained by local psychiatrist - Training adapted to local context by selecting modules based on previous research indicating priority disorders, contextual factors, and participant backgrounds | | Both   - *Quantitative:* Knowledge test before and after training - *Qualitative:* In-depth interviews with trainees on mental health attitudes, integration, and perceived barriers and requirements | - Small increase in knowledge (not statistically significant) - PHC staff discussed their experience of the conflict whilst providing healthcare - Priority physical and mental health disorders explored - Barriers and requirements for integration discussed |

*A: Does the study reference evidence of innovation effectiveness? (N = No, G = Training guidelines referenced only, Y = Yes). B: Does the study reference evidence of innovation cost-effectiveness? (N = No, G = Training guidelines referenced only, Y = Yes). C: But the authors reference a paper that estimates cost of scale up*

##

## Appendix S4 – Full Table of Reported Barriers and Facilitators

Factors that were expected to facilitate scale up or were recommended to overcome a barrier but were not implemented or evaluated in the study are indicated by “*anticipated”.*

|  |  | **Barriers** | **Facilitators** |
| --- | --- | --- | --- |
| **INNOVATION** | |  |  |
|  | Baingana, 2011 | No activities targeted children despite demand | Combining medical / clinical activities with social activities |
|  | Humayun, 2017 |  | Multidisciplinary assessments to avoid purely biomedical approach / over-medicalisation  *Anticipated:*  Introduce livelihood intervention |
| **RESOURCE TEAM** | |  |  |
|  | Boothby, 2011 |  | Availability for ongoing technical assistance for maintenance phase |
|  | Humayun, 2017 | Small group of volunteers, not institutional or government led |  |
|  | Shackman, 2013 | Inadequate sponsor oversight and support where trained service providers were lacking | *Anticipated:*  Clearer strategic goals with realistic and measurable objectives reflecting community needs  Additional oversight |
| **USER ORGANISATIONS** | | |  |
|  | Baingana, 2011 | Agreements not honoured e.g., medication supplies  Attrition of government health workers trained by project |  |
|  | Boothby, 2011 | Mental health staff: outreach and surveillance is absent; poor diagnosis and treatment; high turnover at district clinics and frequent staff absence  Village level mental health cadres often unavailable and lacking contact with service users | Extraordinary efforts by staff |
|  | Budosan, 2011^A^ | NGOs prioritised own needs over MHPSS delivery  NGOs concerned about their rights, which impeded consensus on agreements  Output of local organisations not meeting obligations if not monitored | Commitment of PHC staff to implement policy promoting integration |
|  | Budosan, 2011^B^ | PHC staff not financially or professionally motivated to support individuals identified as severe and referred by community workers resulting in dysfunctional referral system and lack of integration |  |
|  | Budosan, 2016 | Changing practice in line with training is challenging for prescribers and non-specialised staff | Trained staff at managerial level |
|  | Echeverri, 2018 | High staff turnover, often due to refugee setting, so high risk of lack of transmission of knowledge and experience |  |
|  | Hijazi, 2011 | Doctors not changing previous prescribing practice despite training |  |
|  | Humayun, 2017 | Challenge to introduce psychotherapeutic interventions to primarily biomedically trained participants  Reluctance of staff to invest required time for psychological work  Humanitarian actors as psychosocial field staff is unsustainable  High security restrictions in the camps | Trainers were qualified psychiatrists  Experienced professionals in the field was valuable in setting practice trends and providing supervision  *Anticipated:*  Coordination between agencies including district health offices, academic departments of psychiatry and humanitarian agencies |
|  | Jordans, 2016 | Healthcare staff report high distress from additional tasks | NGO’s extensive experience in community psychosocial interventions locally supports feasibility of new mental health cadre  Healthcare staff content with the care being delivered |
|  | Shackman, 2013 | One NGO failed to implement livelihood intervention  Management staff lacked skills for report writing, liaising with agencies, representing at senior level and lacked will to document achievements  Tensions between and within partner organisations - accusations of discrimination, corruption, favouritism, misappropriation of funds, and other misconduct  Traditional healers and health workers lack collaboration: health workers resistant to referring | Traditional healers unthreatened by medical services, more likely to disseminate knowledge to others, and more open to learning than health workers  Audit and address staff skills through supervision, training and mentoring |
|  | Siriwardhana, 2016 | Lack of interest from PHC staff at recruitment or training phases  Low participant retention |  |
| **ENVIRONMENT** | |  |  |
| POLICY / POLITICS | |  |  |
|  | Baingana, 2011 | Mental health medications not included in Essential Drugs Kit |  |
|  | Boothby, 2011 | Mental health not prioritised by Ministry of Health | Advocacy to establish mental health national legislation supporting care continuum |
|  | Budosan, 2011^A^ | Lack of policy promoting integration of mental health into PHC  Lack of policy implementation  Lack of political will | The existence or formulation of a policy promoting integration of mental health into PHC  Political will of government |
|  | Budosan, 2011^B^ | Mental health not prioritised by government |  |
|  |  | Lack of regulations necessitates case-by-case agreements with NGOs |  |
|  | Humayun, 2017 | Lack of government regulation of medication supplies  Lack of political will to invest in programme continuation  Disproportionate resources compared to the population affected | *Anticipated:*  Robust advocacy and collaboration at policy level  Mental health emergency preparedness / disaster plans |
|  | Jordans, 2016 |  | Training curriculum certificates co-issued by Ministry of Health and Population, and recognised by official regulatory body to provide PHC staff mandate  *Anticipated facilitators / recommendations:*  Policy developments promoting scale up of mental health care and staff |
|  | Sadik, 2011 | Contextual challenge of continued conflict in which policy makers operate | Programme integrated into Ministry of Health strategy and policy documents  Ministry of Health funding and expanding training nationally |
|  | Shackman, 2013 | Lack of formal ownership from government impedes sustainability  Stigmatising attitudes reflected at national level through lack of prioritisation and resources and delay in adoption of mental health policy | *Anticipated:*  Recently adopted mental health policy outlined plan to improve access to services, including integration of mental health, decentralisation and community participation, psychiatric medications included in the essentials medicines list, and development of a mental health curricula for general and specialised staff  Contribute to and be guided by government policy |
|  | Siriwardhana, 2016 | Lack of policy for mental health in PHC settings  Lack of coordination between national policy, training programmes and service expansion  Restricted movement and security issues during first post-conflict election | Support from Ministry of Health  *Anticipated:*  Develop uniform guidelines and policies  Integrate mental health strategies into national non-communicable disease prevention plans and post-graduate training using accessible teaching methods e.g., online |
| BUREAUCRACY | |  |  |
|  | Boothby, 2011 | Lack of awareness of benefits among senior policy makers and Ministry of Health officials | *Anticipated:*  Engagement with Ministry of Health staff resistant to decentralisation and involvement of cadres, to support district and village levels  Senior policy makers and Ministry of Health officials made aware of benefits of multilevel decentralised mental health system and replication opportunities for replication  Will to maintain and replicate at all levels  The model perceived as possible in other provinces |
|  | Budosan, 2007 | Disagreement over best approach for national mental health strategy | Discussions to resolve contrasting approaches resulted in development of a national mental health strategy endorsed by parliament stating mental health services should be provided at community level |
|  | Budosan, 2011^B^ | Lack of cooperation from Ministry of Public Health and Population and local health authorities  Lack of administrative unit within the MPHP for mental health | *Anticipated:*  Recent appointment of a mental health representative within Ministry of Public Health and Population anticipated to improve stakeholder coordination |
|  | Budosan, 2016 |  | Main stakeholders of government, health authorities and providers highly motivated to improve services |
|  | Echeverri, 2018 | Insufficient involvement of local and central government health staff | Participation of Ministry of Health deputy director in training session catalysed implementation at refugee and national level, resulting in development of mental health units in general hospitals |
|  | Humayun, 2017 |  | Partnership with military authority who had strong influence over crisis zone helped to overcome barrier of underprioritised mental health in the health system and by the district health department |
|  | Sadik, 2011 |  | Present minister is a psychiatrist |
|  | Siriwardhana, 2016 | Key decision makers at various levels took considerable effort to engage and caused delays | Support from provincial and regional administrative bodies and hospital administrators |
| HEALTH SECTOR | |  |  |
|  | Baingana, 2011 | Lack of transport for breadth of catchment area  Lack of medication due to government not honouring agreement |  |
|  | Boothby, 2011 | *Barriers from district where implementation failed:*  Remote location historically beyond reach of health system, exacerbated by the road being washed away  Medication and treatment only available at district level  Saturation of NGOs possibly undermined collective self-reliance and volunteerism  *Barriers from other districts:*  Coordination lacking between district and subdistrict levels e.g., discharge information  Subdistrict health clinics cover wide geographical area, making follow-up challenging  Mismatch of role expectations between supervisors and staff e.g., community staff required to use own time, transportation and funds for home visits | *Anticipated:*  A psychiatrist to consult on medication a few times a year at subdistrict level to improve attendance and regimes  Motorcycles, fuel and general support for community staff  Education for supervisors on necessity of home visitation  Stricter adherence to two-way referral system to benefit system functioning and continuum of care e.g., discharge information to subdistrict level |
|  | Budosan, 2007 | Lack of permanent local psychiatrists to supervise, particularly in rural disaster-affected areas, requiring others to travel further  Time constraints and workload for delivery agents resulted in insufficient contact time  Shortages of psychiatric medications in the field made on the job training challenging  Layered public health system / dispersal of key players delayed strategic consensus | Recognition of specialisation of delivery agents overtime to support them to restructure time allocation |
|  | Budosan, 2011^A^ | Separate budget allocations  Lack of pre-existing community mental health care | Supervision by psychiatrist |
|  | Budosan, 2016 | Lack of time for staff resulted in improper history taking and examination, comorbidities being missed, and incomplete care |  |
|  | Chandrasiri, 2015 | Lack of human resources to provide adequate services  Inadequate transportation provided to community staff, requiring use of their own means | Pre-existing administrative and supervisory capabilities of government health staff |
|  | Echeverri, 2018 | Refugee settings challenged effective stakeholder coordination  Scarcity of specialised staff limited continued capacity building and posed a barrier for supervision, particularly due to travel distance and cost |  |
|  | Hijazi, 2011 | Lack of pre-existing referral system between primary and secondary services  Head of clinics face several challenges – shortage of staff, inadequate working conditions, limited budget and resources  The challenges posed to heads of clinics resulted in them not always promoting integration into PHC, posing a challenge to trainees |  |
|  | Humayun, 2017 | Mental health not prioritised within the health system  Under-resourced PHC system impedes meaningful integration  Most interventions delivered by doctors – challenge to engage an inclusive team  Dearth of permanent community services, restricting referral opportunities  Dearth of mental health resources locally and large geographical distance  Lack of institutional initiative  Lack of qualified professionals  Poor existing prescribing practices  High workload for staff with minimal breaks  Poor existing knowledge and skill for child and adolescent mental health  Existing resources not adequately trained to deliver interventions according to guidelines  Lack of sustained efforts to reinforce training of staff | Partnership with local department of psychiatry who were aware of need to engage PHC staff  Advocacy efforts prompted provincial government to create additional psychiatrist position  Helpline for GPs established by department of psychiatry to assist referral system  *Anticipated:*  Establish / strengthen PHC in order to facilitate integration  Robust advocacy and collaboration at service level  Paradigm shift in psychiatric departments nationally towards public health  Short term rotations in absence of local specialists |
|  | Jordans, 2016 | Overburdened PHC staff  Insufficient mandate for PHC staff to perform mental health care  Unavailability of medications prescribed by specialists  Lack of private facilities  Additional time required of staff to provide mental health care is burdensome  Community staff unable to terminate treatments | Scope of focussed support interventions delivered by health workers further reduced to only key ingredients such that community counsellors are the only cadre delivering full protocols |
|  | Sadik, 2011 | Contextual challenge of continued conflict in which physicians have to operate |  |
|  | Shackman, 2013 | Multiple NGOs arrived during and after war, independent from government oversight with short-term programmes  Staff received different trainings from NGOs with inconsistent outcomes  Few medical personnel sufficiently trained to diagnose and prescribe  Lack of adequate pay or job security due to project lengths  Community services adversely impacted when volunteers leave for paid positions  Lack of reliable resources including medication and facilities  Stigma among health professionals, reflecting attitudes at national level - lack of training, lack of resources, restriction of mediation access in the provinces |  |
|  | Siriwardhana, 2016 | Lack of support from administrative bodies  High turnover off staff from transfers / rotations caused challenges for participant retention  Financial constraints of PHC staff impeded recruitment  Poor existing diagnosis and treatment provision for MH in PHC  Increased patient loads and lack of human resources impeded training attendance (no replacement staff) and study activities  Trained and specialist human resources are lacking and geographically dispersed  Awareness of mental health problems among PHC staff is low |  |
| SOCIOECONOMIC / CULTURAL | | |  |
|  | Baingana, 2011 | Weak social support system | Involvement of village teams is critical for community mobilisation and sensitisation in lieu of social support |
|  | Boothby, 2011 | *Barriers from the district where implementation failed:*  Ethnically and linguistically unique areas are more isolated  Poor family support, including use of restraints and stigma possibly due to beliefs about causation  Conflict between people with Axis I disorders and community  Failure to discuss mental health beliefs and challenge stigma  *Barriers from other districts:*  Villagers reluctant to speak to researchers or nurses because viewed as outsiders | Community nurses and village cadres introduced or increased awareness of scientific model while remaining sensitive to local explanatory models  Symbiotic relationship between community nurses and village cadres reinforced their respective roles: cadres identified and trusted by community, bridging subdistrict and village levels of care; cadres helped community nurses reach communities that might have considered them as outsiders; nurses add credibility to cadres’ work. |
|  | Budosan, 2007 | Service users unaccustomed to seeking care from Divisional Medical Officers of Health (delivery agents) for mental health problems  “Western”, goal-oriented style of INGOs, tight deadlines and funding constraints clashed and frustrated personal qualities of host population | Patience, endurance and self-control qualities of host population was a pre-requisite to successful implementation, in contrast to default “western” style of INGOs |
|  | Budosan, 2016 |  | Community stakeholder support and participation |
|  | Chandrasiri, 2015 | Stigma and discrimination |  |
|  | Echeverri, 2018 | Language barriers  Underrepresentation of women among community workers confounded the cultural restraint for female service users to talk to men about mental health problems |  |
|  | Humayun, 2017 | Ethnic / religious minorities within camps not accessing services without dedicated outreach  Security restrictions in camp restricted travelling after dark  Language, dialect and culture barrier to delivering psychological interventions  Women reluctant to talk to male workers, seek help, or uncover face during consultation, and many (especially men) unable to discuss emotional difficulties  Close family / key informants not always available to offer collateral accounts |  |
|  | Jordans, 2016 | Stigma  Low demand for mental health care |  |
|  | Shackman, 2013 | Lack of social and cultural aspects of mental health and contextually appropriate treatments  Social, community and family support and work neglected in favour of individual approach |  |
|  | Siriwardhana, 2016 | Stigma prevented treatment seeking  Community awareness is low |  |
| PEOPLE’S NEEDS & RIGHTS | | |  |
|  | Budosan, 2007 |  | Confidentiality was prioritised |
|  | Budosan, 2011^A^ |  | Respect for patient confidentiality |
|  | Chandrasiri, 2015 | Lack of financial help for service users / families  Poor economic status, remote living and inadequate public transport services made travel for service users challenging  Service users have to adjust at programme completion to handing over of responsibilities to the Ministry of Health |  |
|  | Jordans, 2016 | Attrition rates for service users is generally high, particularly among those with alcohol use disorders, and includes the following reasons: medication side effects, time constraints, the belief that they do not have a mental health problem, and distance |  |
|  | Shackman, 2013 | Distance and transport challenges result in financial, time, and access consequences for service users and families  Alcohol and substances more available and accessible since the war, and increased use reportedly due to peer pressure and unemployment – unclear to what extent drug abuse is leading to mental illness or what effective treatments might help  Service users rarely attributed problems to conflict, instead reporting cultural traditional explanations or situational factors such as frustration or disappointment  Many reluctant to take medication and lacked family support, with high relapse rates  Need to go beyond 'trauma related issues pertaining to the war' and towards stigma, education, and culturally appropriate community and social support for individuals and families |  |
|  | Siriwardhana, 2016 | Lack of existing epidemiological data about burden in PHC |  |
| **SCALING UP STRATEGY** | | |  |
| DISSEMINATION | |  |  |
|  | Baingana, 2011 | No refresher training resulted in attrition of personnel, knowledge and skills  Weak supervision  Insufficient number of village workers trained | Regular (quarterly) coordination and joint planning between NGO and government  Support supervision is critical |
|  | Budosan, 2007 | On the job training was challenging due to lack of mental health experience | Refresher practical training during theoretical training overcame some barriers for on the job training  Collaboration with other services to avoid duplication and overloading of staff post-disaster  Strategic selection of delivery agent as per their request for training and will to train own staff  Training met the three requirements of effective educational interventions, followed consensus-based guidelines and was grounded in respect and community involvement  Training was culturally sensitive and appropriate for the context – especially important for on the job training  Training facilitated dialogue between trainers, trainees and other stakeholders to integrate perspectives |
|  | Budosan, 2011^A^ |  | Cohesion between all stakeholders  Open dialogue between trainers, trainees and other mental health players  Professional training in line with consensus guidelines combining theoretical and on-the-job elements  Training that is ongoing, participatory and integrated within clinical context  Culturally and contextually appropriate training |
|  | Budosan, 2011^B^ | Change in knowledge did not result in change in practice which impeded the establishment of a working referral system |  |
|  | Budosan, 2016 |  | Collaboration within and beyond the health sector  *Anticipated:*  Strengthening inter-sectoral collaboration at local level |
|  | Chandrasiri, 2015 |  | Experts delivered training and continued to contribute to capability enhancement  Selection of trainees; highly motivated, available, experienced and integrated into the community |
|  | Echeverri, 2018 | Inadequate selection of trainees – too heterogeneous in terms of knowledge levels and profiles, underrepresentation of women among community workers | Involvement of local psychiatrists in training assisted with improved quality and establishment of referral systems  Train-the-trainers and supervisors model allowed trainers to consolidate training, gain confidence, and create links with general health staff, enhancing collaboration, supervision and sustainability in lieu of specialists  Joint training with community, clinician, and managerial staff improved multilevel collaboration and understanding of respective roles  Establishing links with traditional healers enhanced referrals  *Anticipated:*  Knowledge and skill transfer and refresher and continued training to combat high staff turnover of refugee settings  Building mental health capacity in nurses could combat lack of specialist staff to continue capacity building and supervision, as they are often more available in refugee settings and rotate less often  Peer support, online linkages and telephone assistance should be considered |
|  | Hijazi, 2011 | Lack of continued support and supervision post-training | Tailored training to different staff groups to facilitate team approach to care  Conducting training for separate staff groups facilitated open discussion  Piloted integration of case management teams for PHC clinics  Informal networks established between trainers and trainees for future technical input on cases  Map mental health services within clinic areas to assist trainees in referral pathways  *Anticipated:*  Strengthen consultation and communication among trainees and specialists post-training by strengthening referral networks and supervision  Orientation day for heads of clinics, focussing on importance of integration, to determine roles of PHC workers, and explore their roles in integration |
|  | Humayun, 2017 | Training contained overly complex principles, lack of examples, and not enough emphasis on potential harms e.g., medicating children  Absence of refresher training  Task-shifting is time-consuming initially | Task-shifting allowed specialists to prioritise severe presentations and delegation, and provided them with valuable training opportunities  Use travelling time to discuss clinical needs and developing skills  Adapted to biomedical approach of trainees by grouping psychosocial interventions as ‘counselling’ and not distinguishing between different techniques  *Anticipated:*  Robust advocacy and collaboration at training level |
|  | Jordans, 2016 |  | Community workers and mother groups trained in community informant detection tool to overcome demand-side barrier  Community staff emphasising family support interventions involves families more  Manualised protocols made stricter to assist counsellors with treatment termination, avoid capacity problem and prepare for further scale up  New duties delegated between and within staff groups to reduce overburdened roles |
|  | Shackman, 2013 | Distance and transport challenges for attending training and learning from outreach services  Distance and transport challenges restricted selection criteria of trainees – prioritised accessibility over suitable candidates  Separate trainings for medical and traditional healers  Advanced and westernised counselling techniques and biomedical curriculums presented and not followed up to determine effectiveness  Trainees rarely contacted after workshops despite expressing interest  Learning was not reinforced, and no support available for ongoing challenges | Training, direct services and outreach reduced stigma among staff  Radio programmes successful for disseminating to community members  *Anticipated facilitators / recommendations:*  Integrating groups at training and improving understanding respective skills, expertise and approaches  Recently adopted training needs to be adapted to the context |
|  | Siriwardhana, 2016 | Training not locally relevant enough – specifically not addressing culture and conflict-related experiences  Lack of culturally and contextually appropriate training materials | Adapt to operational challenges  Delivered training to maximum PHC staff in region to generate interest among PHC and administrative bodies about initiative  Group activities and role plays were positively received  Engaged doctors union and involved central and provincial health authorities to mitigate lack of interest among PCP  *Anticipated:*  Effectiveness of training enhanced by using local language and culturally and contextually relevant materials  Shorter training sessions with refresher sessions and continuous monitoring and support |
| ORGANISATIONAL CHOICES | | |  |
|  | Baingana, 2011 | NGO outreach clinics linked to IDP camps - access to services will become more challenging as population returns to original villages |  |
|  | Boothby, 2011 |  | Addressing multiple levels of the care continuum  *Anticipated*:  Additional resources to maintain quality of advanced-level nurse training and staffing ratios required for population  Prioritise retention of district-level psychiatrist beyond initial six months and continued decentralisation of medication provision |
|  | Budosan, 2011^B^ |  | Integrated rather than vertical services perceived as more sustainable, effective and preparatory for new emergencies |
|  | Chandrasiri, 2015 |  | Organisational structure of programme on existing health care structure of the country and linking with PHC  Outreach programme partially overcame human resource issue  Establish supervision timetable |
|  | Echeverri, 2018 | Not including key staff from different levels negatively impacted establishment of referral system | Public and sector partnerships at local and central level is a key strategy for sustainability |
|  | Humayun, 2017 | Due to environmental barriers, services had to be located at tertiary hospital, therefore access remained a barrier for many | Simplifying and reprioritising innovations and trainings for the population at hand |
|  | Sadik, 2011 |  | Embedding the programme into the health system promotes sustainability |
|  | Shackman, 2013 | Locating trainings and trainees in regional towns impedes community access to staff and awareness raising |  |
|  | Siriwardhana, 2016 |  | *Anticipated*:  Train other staff groups (public health staff, midwives, hospital staff, social workers) to enhance community outreach, increase awareness, reduce stigma, increase detection |
| COST/RESOURCE MOBILISATION | | |  |
|  | Baingana, 2011 |  | Small amount of funds provided initially for transportation, coordination, and medication |
|  | Boothby, 2011 | Funding waned after emergency response phase – no funding for development | The tsunami presents an opportunity (international and national funds and interest) to make systematic changes exceeding pre-crisis services  *Anticipated:*  Strong national advocacy campaign for funding  International agencies to continue to support programme and coordinate awareness raising |
|  | Budosan, 2011^A^ |  | Opportunistic timing – influx of funding and expertise post-disaster |
| MONITORING AND EVALUATION | | |  |
|  | Baingana, 2011 | Inconsistencies between activities and reports  Lack of documentation of training |  |
|  | Boothby, 2011 | *Barriers from district where implementation failed:*  Lack of valid data i.e., inconsistencies between activities and reports |  |
|  | Budosan, 2011^A^ | Lack of mental health research on long-term outcomes and integration of mental health into PHC more generally |  |
|  | Budosan, 2011^B^ |  | Needs and service assessment before scale up informed design for local context |
|  | Budosan, 2016 | Lack of standardised forms |  |
|  | Chandrasiri, 2015 |  | Organised and comprehensive information system, particularly on community staff performance monitoring, summated and interpreted at divisional and regional levels  Continuous progress review mechanism: continuity and technical support by expert for review process was key |
|  | Echeverri, 2018 | Evaluation tools not adapted to staff profiles - reliability and validity issues of evaluation methods raised by trainers |  |
|  | Hijazi, 2011 | Lack of pre-existing routine mental health service user data  Outpatient record forms not regularly used  Tracking and reporting requires institutional agreement, organisational change and commitment | Formative evaluations conducted throughout to adapt to needs  *Anticipated:*  Work with heads of clinics to advocate for and support integration at all levels including report taking |
|  | Humayun, 2017 | No evaluation of impact of training on identification and management of mental health problems or referrals  Absence of Health Information System  Documentation sometimes impossible due to workload / demand, resulting in missing data |  |
|  | Jordans, 2016 | Record keeping not always accurate |  |
|  | Shackman, 2013 | Paper or journal books made storage and retrieval of records challenging for providers and administrators  Minimal M&E by partners - annual reports for funding agencies often not updated nor communicating needs / priorities, key documents were lost, including training course curricula, making cross checking difficult  Data collected was not incorporated into programme planning or evaluation, e.g., resources allocated and surveys completed for morbidity surveys but results not valued or used | *Anticipated:*  More practice-based research to determine how to adapt interventions  Realistic, workable and acceptable system of documentation and communication, and method for duplication and back up |
|  | Siriwardhana, 2016 |  | *Anticipated:*  Data on existing burden in PHC can inform integration |

# Appendix S5: Quality Appraisal of Included Studies using the Mixed Methods Quality Appraisal Tool (MMAT)

|  | |  |  |  |  |  |  |  |  |  |  |  |  |  |  |  |
| --- | --- | --- | --- | --- | --- | --- | --- | --- | --- | --- | --- | --- | --- | --- | --- | --- |
|  |  | Baingana, 2011 |  | Boothby, 2011 | Budosan, 2007 | Budosan, 2011 | Budosan, 2011 | Budosan, 2016 | Chandrasiri, 2015 | Echeverri, 2018 | Hijazi, 2011 | Humayun, 2017 | Jordans, 2016 | Sadik, 2011 | Shackman, 2013 | Siriwardhana,2016 |
| Screening Questions | |  |  |  |  |  |  |  |  |  |  |  |  |  |  |  |
|  | S1. Are there clear research questions? | Y |  | Y | Y | Y | Y | Y | Y | Y | Y | Y | Y | Y | Y | Y |
|  | S2. Do the collected data allow to address the research questions? | Y |  | Y | Y | Y | Y | Y | Y | Y | Y | Y | Y | Y | Y | Y |
| Qualitative Studies | |  |  |  |  |  |  |  |  |  |  |  |  |  |  |  |
|  | 1.1. Is the qualitative approach appropriate to answer the research question? | CT |  | Y | Y | Y | Y | - | Y | Y | Y | - | Y | - | Y | Y |
|  | 1.2. Are the qualitative data collection methods adequate to address the research question? | CT |  | Y | CT | Y | Y | - | CT | Y | Y | - | Y | - | Y | Y |
|  | 1.3. Are the findings adequately derived from the data? | CT |  | Y | CT | CT | CT | - | CT | CT | CT | - | Y | - | Y | Y |
|  | 1.4. Is the interpretation of results sufficiently substantiated by data? | CT |  | Y | CT | CT | CT | - | CT | CT | CT | - | Y | - | Y | Y |
|  | 1.5. Is there coherence between qualitative data sources, collection, analysis and interpretation? | CT |  | CT | CT | CT | Y | - | CT | CT | CT | - | Y | - | Y | Y |
| Non-randomised studies | |  |  |  |  |  |  |  |  |  |  |  |  |  |  |  |
|  | 3.1. Are the participants representative of the target population? | - |  | Y | CT | Y | Y | - | - | Y | Y | Y | - | Y | - | Y |
|  | 3.2. Are measurements appropriate regarding both the outcome and intervention (or exposure)? | - |  | Y | Y | Y | Y | - | - | CT | Y | Y | - | Y | - | Y |
|  | 3.3. Are there complete outcome data? | - |  | Y | N | N | CT | - | - | CT | Y | Y | - | Y | - | Y |
|  | 3.4. Are the confounders accounted for in the design and analysis? | - |  | N | CT | CT | CT | - | - | CT | CT | CT | - | CT | - | Y |
|  | 3.5. During the study period, is the intervention administered (or exposure occurred) as intended? | - |  | Y | CT | CT | CT | - | - | CT | Y | CT | - | CT | - | Y |
| Quantitative Descriptive Studies | | - |  |  |  |  |  |  |  |  |  |  |  |  |  |  |
|  | 4.1. Is the sampling strategy relevant to address the research question? | - |  | Y | Y | CT | Y | Y | Y | CT | Y | - | Y | - | - | - |
|  | 4.2. Is the sample representative of the target population? | - |  | Y | CT | CT | Y | Y | Y | CT | Y | - | Y | - | - | - |
|  | 4.3. Are the measurements appropriate? | - |  | Y | Y | CT | Y | Y | CT | CT | Y | - | Y | - | - | - |
|  | 4.4. Is the risk of nonresponse bias low? | - |  | CT | CT | CT | CT | CT | CT | CT | Y | - | Y | - | - | - |
|  | 4.5. Is the statistical analysis appropriate to answer the research question? | - |  | Y | NA | NA | NA | NA | NA | NA | NA | - | NA | - | - | - |
| Mixed Methods Studies | |  |  |  |  |  |  |  |  |  |  |  |  |  |  |  |
|  | 5.1. Is there adequate rationale for using a mixed methods design to address the research question? | - |  | Y | N | Y | N | - | N | - | N | - | Y | - | - | - |
|  | 5.2. Are the different components of the study effectively integrated to answer the research question? | - |  | Y | Y | N | Y | - | Y | - | Y | - | Y | - | - | - |
|  | 5.3. Are the outputs of the integration of qualitative and quantitative components adequately interpreted? | - |  | Y | CT | CT | Y | - | CT | - | CT | - | Y | - | - | - |
|  | 5.4. Are divergences and inconsistencies between quantitative and qualitative results adequately addressed? | - |  | Y | CT | CT | CT | - | CT | - | Y | - | CT | - | - | - |
|  | 5.5. Do the different components of the study adhere to the quality criteria of each tradition of the methods involved? | - |  | Y | CT | CT | CT | - | N | - | Y | - | Y | - | - | - |

*Y = Yes. CT = Cannot Tell. N = No. NA = Not Applicable*

*Note:* As no studies involved randomised controlled trials, section two of the MMAT has not been displayed
